# Establishing an *In Vivo* Assay System to Identify Components Involved in Environmental RNA Interference in the Western Corn Rootworm

**DOI:** 10.1371/journal.pone.0101661

**Published:** 2014-07-08

**Authors:** Keita Miyata, Parthasarathy Ramaseshadri, Yuanji Zhang, Gerrit Segers, Renata Bolognesi, Yoshinori Tomoyasu

**Affiliations:** 1 Department of Biology, Miami University, Oxford, Ohio, United States of America; 2 Biotechnology Division, Monsanto Company, Chesterfield, Missouri, United States of America; Kansas State University, United States of America

## Abstract

The discovery of environmental RNA interference (RNAi), in which gene expression is suppressed via feeding with double-stranded RNA (dsRNA) molecules, opened the door to the practical application of RNAi-based techniques in crop pest management. The western corn rootworm (WCR, *Diabrotica virgifera virgifera*) is one of the most devastating corn pests in North America. Interestingly, WCR displays a robust environmental RNAi response, raising the possibility of applying an RNAi-based pest management strategy to this pest. Understanding the molecular mechanisms involved in the WCR environmental RNAi process will allow for determining the rate limiting steps involved with dsRNA toxicity and potential dsRNA resistance mechanisms in WCR. In this study, we have established a two-step *in vivo* assay system, which allows us to evaluate the involvement of genes in environmental RNAi in WCR. We show that *laccase* 2 and *ebony*, critical cuticle pigmentation/tanning genes, can be used as marker genes in our assay system, with *ebony* being a more stable marker to monitor RNAi activity. In addition, we optimized the dsRNA dose and length for the assay, and confirmed that this assay system is sensitive to detect well-known RNAi components such as *Dicer-2* and *Argonaute-2*. We also evaluated two WCR *sid1- like* (*sil*) genes with this assay system. This system will be useful to quickly survey candidate systemic RNAi genes in WCR, and also will be adaptable for a genome-wide RNAi screening to give us an unbiased view of the environmental/systemic RNAi pathway in WCR.

## Introduction

RNA interference (RNAi) is an evolutionarily conserved mechanism, in which double-stranded RNA (dsRNA) molecules trigger gene silencing in a sequence specific manner [1]–[3]. The discovery of RNAi has revolutionized many fields of biology by allowing loss-of-function analyses in various organisms without laborious and time-consuming genetic manipulations [4]. RNAi has also provided a promising new trend to the pest management field [5], as RNAi-based pest control strategies have the potential to target pest species with great specificity. However, application of RNAi to pest control is still a challenge in part due to the difficulty of effectively delivering dsRNA molecules into organisms [5]. Interestingly, in some organisms including several pest insects, RNAi can be triggered via feeding with dsRNA molecules (feeding RNAi or environmental RNAi), and RNAi in these organisms often works systemically as well (for example, see [6]–[9]. Also see [5] for review). The ease of inducing a systemic RNAi response via dsRNA feeding opens the door to the practical application of RNAi-based techniques in crop pest management.

Although there are some variations, the core RNAi components, such as Argonaute (Ago) and Dicer (Dcr), are well conserved among taxa (reviewed in [2], [3]). In contrast, the ability of an organism to display a robust systemic RNAi response varies greatly among organisms [4], [5], [10]–[12]. Systemic RNAi can be categorized into several separable processes, including (i) the cellular uptake of dsRNA from the extracellular environment, and (ii) the spreading of the silencing signal between cells. In addition, (iii) intestinal dsRNA uptake is an essential step in RNAi triggered by feeding with dsRNA molecules (environmental RNAi). The environmental RNAi can be restricted to the intestinal cells, or works systemically with the help of the first two steps. Some organisms display all three systemic RNAi responses (allowing environmental RNAi works systemically), while others show only a few or none. The determining factors causing the differences in the ability to elicit a robust systemic RNAi response among organisms are currently unknown (see [12] for review). In silkmoth (Lepidoptera), reduction in the expression of two RNAi genes, *R2D2* and *Translin*, appears to be a contributing factor for the lack of the robust RNAi response [13].

The molecular basis of systemic RNAi in animals has been studied most extensively in a nematode, *Caenorhabditis elegans* [10], [14]. These studies have identified a battery of genes critical for systemic RNAi in *C. elegans*, including *sid-1* [15], [16]. *sid-1* codes for a dsRNA channel, indicating that a channel-based dsRNA transport is an essential mechanism in the *C. elegans* systemic RNAi response [15], [16]. Endocytosis also appears to be crucial in systemic RNAi in *C. elegans* [14], [17], as an inhibition of endocytosis components represses systemic RNAi. Although some insects also exhibit a robust systemic RNAi response, the extent of the conservation of these systemic RNAi components in insects is still elusive. Dipteran insects, such as flies and mosquitos, lack *sid-1* homologs in their genomes [11]. Since *Drosophila* also lack a robust systemic RNAi response [18], the correlation between the presence of a robust systemic RNAi response and the presence of the *sid-1* homologs (*sid-1 like* genes, *sil* [11]) has been proposed [16]. However, this correlation has been challenged ([11], also see Discussion for details). In addition to the Sid-1-based dsRNA transport, the involvement of endocytosis has also been reported in insect systemic RNAi by using a *Drosophila* cell culture system [19], [20]. Nevertheless, the molecules and pathways involved in systemic RNAi in insects remain largely unknown.

The western corn rootworm (WCR, *Diabrotica virgifera virgifera*) is one of the most devastating corn pests in North America, causing yield losses that are estimated to exceed US$1 billion annually. In the recent years, corn rootworm populations have evolved to resist chemical insecticides as well as cultural control practices [21], [22]. Furthermore, a potential resistance evolution of WCR to the first generation Bt maize crops has been reported [23], making WCR a notorious pest to manage in North America. Interestingly, WCR displays a robust environmental RNAi response [6], raising the possibility of applying an RNAi-based pest management strategy to this pest. WCR appears to be capable of performing above mentioned all three steps of systemic RNAi, as a non-intestinal gene can be silenced via feeding RNAi (which requires intestinal dsRNA uptake, followed by releasing of the silencing signal from intestinal cells and receiving of the signal in other tissues) [24], [25]. A transgenic-based expression of dsRNA in the corn that targets endogenous WCR genes was demonstrated to be effective to suppress the WCR activity [6], showing that RNAi-based pest management is a promising alternative to conventional pesticides.

Understanding the molecular mechanisms involved in the environmental RNAi process in WCR will allow for determining the rate limiting steps involved with dsRNA toxicity in WCR and potential dsRNA resistance mechanisms. Previously, an *in vivo* assay system to screen for genes involved in the RNAi pathways has been reported in another beetle, *Tribolium castaneum* [11]. This assay system was adapted to WCR that will be useful in identifying genes involved in environmental RNAi. The WCR assay system consists of two RNAi feeding experiments; (i) dsRNA for a candidate gene involved in environmental RNAi is fed to WCR larvae for two to three days; and (ii) WCR larvae are fed with dsRNA for a “marker” gene. The marker gene would be a gene that has a visual and/or measurable function in the insect, in which the effect can be easily observed and measured upon knockdown. If the candidate gene in the first step is essential for RNAi (including environmental RNAi), the messenger RNA (mRNA) levels of the marker gene will not be altered by the second RNAi, hence no changes in phenotype will be detected. If, on the other hand, the phenotypic change is observed due to successful knockdown of the marker gene, this will indicate that the candidate gene is not involved in RNAi.

In this study, we first evaluated several genes as potential marker genes, and identified two genes, *ebony* and *laccase 2* (*lac2*), as markers for our assay system. We next evaluated the optimal length and concentration of dsRNA for the assay system, and also investigated the possibility of competition between the first and second RNAi. Interestingly, we noticed that competition occurs not only depending on the concentration of the tested dsRNA molecules but also depending on their lengths of the dsRNAs tested, which may give us a clue to the molecular basis of systemic RNAi. We also utilized two well-known RNAi core genes, *Dcr2* and *Ago2*, as positive controls for this assay system, and confirmed that the assay system is sensitive enough to specifically identify genes involved in the RNA pathway. Finally, we tested two WCR *sil* genes with the assay system. The marker RNAi suppression by *sil* RNAi was significant but not robust, which may suggest a partial involvement of *sil* genes in the WCR environmental RNAi. This assay system will enable the survey of candidate systemic RNAi genes in WCR, and will also be adaptable for a genome-wide RNAi screening to provide us with an unbiased view of the environmental/systemic RNAi pathway in WCR.

## Results

### Identification of marker genes for the *in vivo* assay system

An ideal marker gene would be a gene whose knock-down will result in a clear visible phenotype without affecting larval mortality. We focused on genes that are involved in body color formation, such as *yellow* genes, *laccase* genes, and *ebony* [26]–[28]. *yellow* genes are a group of genes critical for melanin biosynthesis [29]. A mutation in *yellow* in *Drosophila* causes lack of melanin-based pigmentation [29], [30]. RNAi or loss-of-function experiments for *yellow* genes have been performed in other insects, some of which have also resulted in reduction of melanin-based pigmentation [31]–[33]. *laccase* genes code for phenol oxidases (POs). *laccase 2* (*lac2*) has been identified as a critical PO for body wall pigmentation and sclerotization in the red flour beetle, *Tribolium castaneum*, as well as in some other insects including WCR [34], [35]. *ebony* codes for NBAD (N-beta-alanyl dopamine) synthetase, which is critical for the formation of NBAD sclerotin [26], [28]. A mutant or knock down for *ebony* in some insects causes more Dopamine to shunt into the melanin production pathway, resulting in a darker body color [30], [31], [33].

A BLAST search for *yellow* homologs using the WCR unigene database identified six unigene contigs that are similar to the *Tribolium yellow* genes in WCR. We could not identify a unigene orthologous to *yellow-y* (Figure S1), which appears to be a main pigmentation gene in other insects. Nonetheless, we decided to pursue two of the WCR *yellow* homologs (*yellow-f* and *yellow-c*) that are related to *yellow-y* based on the phylogenetic tree (Figure S1), as they may have a similar pigmentation function as *yellow-y* in WCR. In addition, we also identified the WCR orthologs for *lac2* and *ebony* from the WCR unigene database.

**Figure 1:**
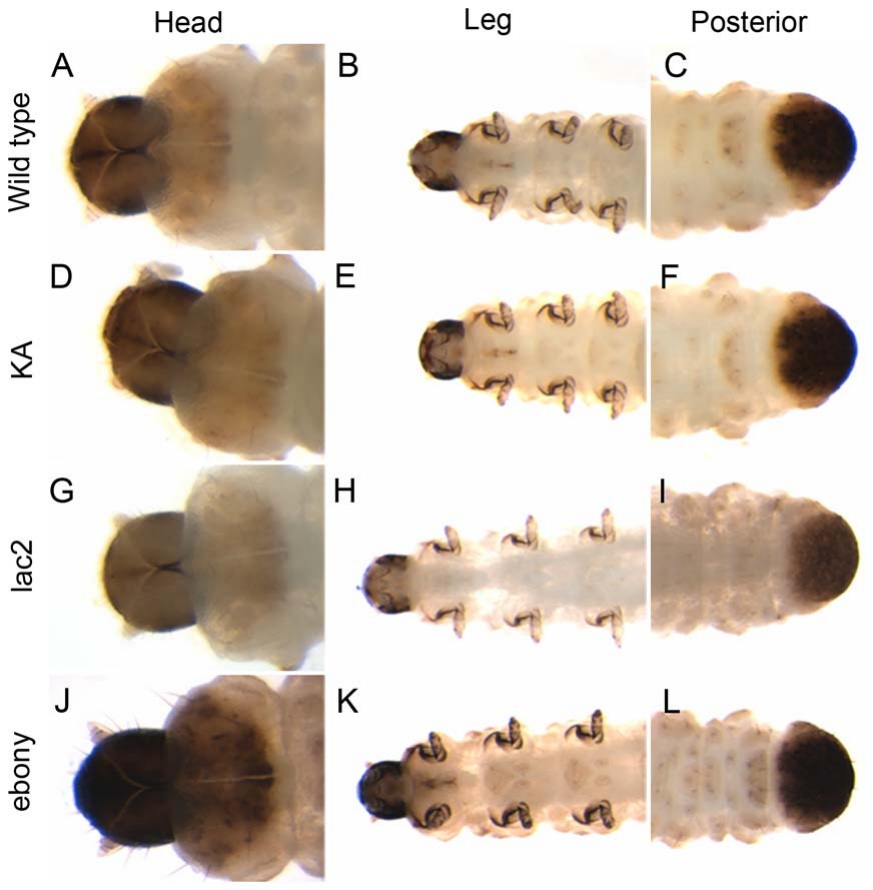
*lac2* and *ebony* feeding RNAi phenotypes in WCR. (A–C) wild-type, (D–F) KA dsRNA fed, (G–H) *lac2* RNAi, and (J–L) *ebony* RNAi. Both *lac2* and *ebony* RNAi affect the pigmentation seen in the larval head, legs, and the posterior-most segment.

We next analyzed the RNAi phenotypes of these genes, and evaluated their potential as a marker gene in the assay system. dsRNA for these genes was fed to first-instar larvae (one day after hatching; DAH) at 5 µg per 1 mL diet (lengths of the dsRNA molecules used are found in Table S1). The body color phenotypes of the resulting larvae were then analyzed at the second larval stage (after the first larval molt), as molting is usually required to affect larval body color via gene depletion. Among the potential marker genes tested, RNAi for *lac2* and *ebony* resulted in visible pigmentation defects (Figure 1). RNAi for *lac2* caused a reduction of the black pigmentation in the head, legs, and the posterior-most segment (Figure 1 G–I). RNAi for *ebony* affected the pigmentation in a similar area as the *lac2* RNAi, but instead induced a stronger dark black pigmentation than controls (Figure 1 J–L). These pigmentation defects were not observed when a mock dsRNA (KA dsRNA) was fed (Figure 1 D–F). Both the *lac2* and *ebony* RNAi phenotypes in WCR are also consistent with the previously reported functions of these genes in other insects [26]–[28], [31], [34], [35]. In contrast to *lac2* and *ebony*, RNAi for the two WCR *yellow* genes did not result in a noticeable pigmentation defect (Figure S2). We tried each single RNAi as well as double RNAi for the two *yellow* genes; however, we did not detect an altered pigmentation phenotype caused by these RNAi treatments despite the reduction of the mRNA level confirmed by qPCR (Figure S3). This result suggests that either these two WCR *yellow* genes are not involved in larval body pigmentation in WCR, or are acting redundantly with an unidentified WCR ortholog of *yellow-y*. Because RNAi for other candidate marker genes resulted in visible pigmentation phenotypes, we decided not to pursue *yellow* genes further for the assay system in WCR. In contrast to the *yellow* gene RNAi, both the *lac2* and *ebony* RNAi resulted in the phenotypes that are visible, with high penetrance, and with no immediate lethality, therefore are suitable for the assay system with regard to scoring reliability.

### Length and dose dependency of feeding RNAi in WCR

**Figure 2:**
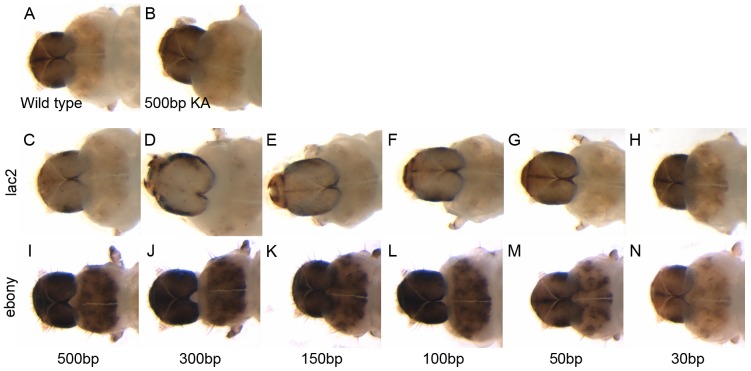
Effect of dsRNA length on feeding RNAi efficiency. (A) wild-type, (B) KA dsRNA, (C–H) *lac2* RNAi with various lengths of dsRNA, (I–J) *ebony* RNAi with various lengths of dsRNA. Note that feeding dsRNA longer than 100 bp induced easily identifiable pigmentation defects both in *lac2* and *ebony* RNAi.

dsRNA length is known to affect the efficiency of the systemic RNAi response, with a longer dsRNA being more efficient to trigger RNAi (though it is currently unknown whether there is a limit to the increased triggering efficiency of a longer dsRNA molecule with direct relation to its length). This dependency was first observed in *C. elegans* [16], [36], and has now also been confirmed in several insects, including *Tribolium castaneum* and WCR [25], [37]. We tested several different lengths of dsRNA for *lac2* and *ebony* to evaluate whether dsRNA length is a significant factor for the assay system. Among the various lengths of *lac2* dsRNA tested, RNAi with dsRNA molecules longer than 100 bp resulted in phenotypes that are easily distinguishable from those of wild-type (Figure 2 C–F). In contrast, The WCR larvae fed with 50 bp or 30 bp *lac2* dsRNA failed to show any noticeable *lac2* RNAi phenotypes (Figure 2 G–H). We also tested the dsRNA length dependency for *ebony* RNAi, and noticed the same tendency, in which the dsRNA molecules longer than 100 bp resulted in recognizable *ebony* RNAi phenotype (Figure 2 I–N). These results indicate that dsRNA longer than 100 bp will be required to induce a recognizable RNAi phenotype in the assay system.

**Figure 3:**
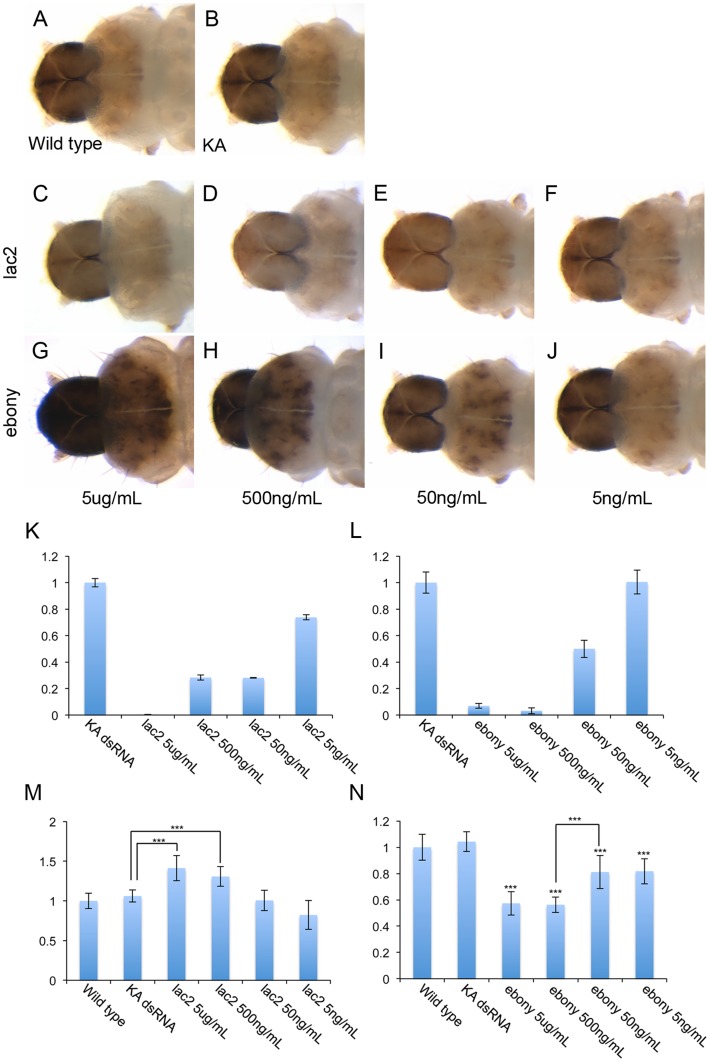
Effect of dsRNA dose on feeding RNAi efficiency. (A) wild-type, (B) KA dsRNA, (C–F) *lac2* RNAi with various doses of dsRNA, (G–J) *ebony* RNAi with various doses of dsRNA. Note that feeding 500 ng/mL or more of dsRNA induced clear pigmentation defects both in *lac2* and *ebony* RNAi. (K, L) Reduction of mRNA by various doses of *lac2* dsRNA (K) or *ebony* dsRNA (L). The Y-axis indicates relative expression levels compared to the control (KA dsRNA). (M, N) quantification of larval head pigmentation by ImageJ. The Y-axis indicates the pigmentation index with 1 being the wild-type mean gray value. The lower the value, the darker the head pigmentation. (M) ImageJ analysis for *lac2* RNAi with various doses of dsRNA. Larvae fed with 5 µg/mL and 500 ng/mL of dsRNA had significantly higher pigmentation values compared to the KA dsRNA control larvae (P***<0.001, n = 10). For an unknown reason, the larvae fed with 5 ng/mL of dsRNA had a lower pigmentation value than that of control. (N) ImageJ analysis for *ebony* RNAi with various doses of dsRNA. All four doses of dsRNA had significantly lower pigmentation indices compared to the KA dsRNA control (P***<0.001, n = 10). Larvae fed with 5 µg/mL and 500 ng/mL of dsRNA had significantly lower pigmentation values compared to the larvae fed with 50 ng/mL of dsRNA (P***<0.001, n = 10).

We also tested the dose dependency of WCR feeding RNAi by using various amount of *lac2* and *ebony* dsRNA. We used a 250 bp dsRNA, at varying concentrations of 5 µg, 500 ng, 50 ng and 5 ng per 1 mL of diet. Quantitative RT-PCR analysis demonstrated that 50 ng/mL of dsRNA is sufficient to induce significant mRNA reduction for both *ebony* and *lac2* (Figure 3 K–L). However, the WCR larvae fed with 50 ng/mL or 5 ng/mL of the dsRNA failed to exhibit a clear pigmentation defect (Figure 3 E–F and I–J), suggesting that these amounts are not sufficient for our assay system. In contrast, the larvae fed with 5 µg/mL and 500 ng/mL of dsRNA resulted in easily recognizable pigmentation defects (Figure 3 C–D and G–H). To analyze the pigmentation phenotypes quantitatively, we measured the intensity of the head pigmentation by ImageJ [38]. For *lac2* RNAi, pigmentation changes caused by 5 µg/mL and 500 ng/mL of dsRNA, but not by 50 ng/mL, were statistically significant (Figure 3 M. P***<0.001, n = 10). For *ebony* RNAi, all dose of dsRNA induced statistically significant changes in head pigmentation (Figure 3 N. P***<0.001, n = 10), however, 5 µg/mL and 500 ng/mL of dRNA of dsRNA induced significantly stronger pigmentation changes compared to that induced by 50 ng/mL (Figure 3 N, P***<0.001, n = 10).

Taken together, these results indicate that dsRNA longer than 100 bp and at a concentration of at least 500 ng/mL is required to have a “scoreable” pigmentation phenotype when *ebony* or *lac2* is used as the marker in our assay system.

### Identification of core RNAi components

**Figure 4:**
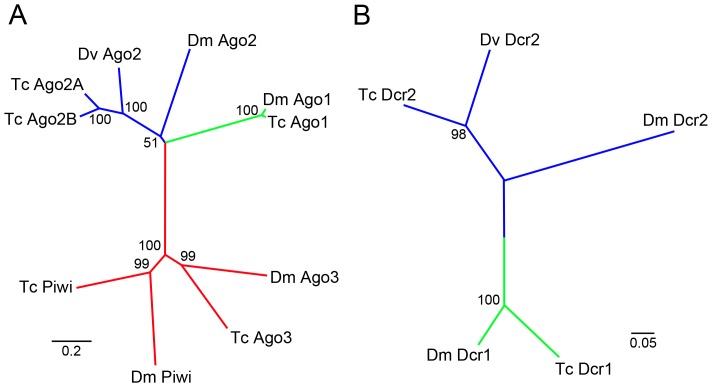
RNAi core components in WCR. (A) phylogenetic analysis for Ago proteins. WCR Ago2 identified in this study appears to be orthologous to both Tc-Ago2A and Ago2B. (B) phylogenetic analysis for Dcr proteins.

Evolutionarily conserved RNAi core genes are good positive controls to assess the efficiency of our assay system. We identified two of the RNAi core component genes, *Ago2* and *Dcr2* from the WCR unigene database (Figure 4). Phylogenetic analysis revealed that *Ago2* we identified from the WCR database appears to be orthologous to both *Tribolium Ago2* paralogs (*Tc-Ago2A* and *Tc-Ago2B*) (Figure 4 A). We performed RNAi for these core RNAi genes and determined whether RNAi for the core genes produce any noticeable phenotype. If RNAi for these RNAi core genes affect larval pigmentation or mortality, we will not be able to use these genes as positive controls. RNAi for *Ago2* and *Dcr2* did induce significant reduction of their mRNA level (Figure S3), however, neither *Ago2* RNAi nor *Dcr2* RNAi resulted in any noticeable abnormalities (data not shown). Body pigmentation of these RNAi larvae was also unaffected. Therefore both *Ago2* and *Dcr2* are useful as positive controls for the assay system in WCR.

### WCR life cycle and the assay system time course

**Figure 5:**
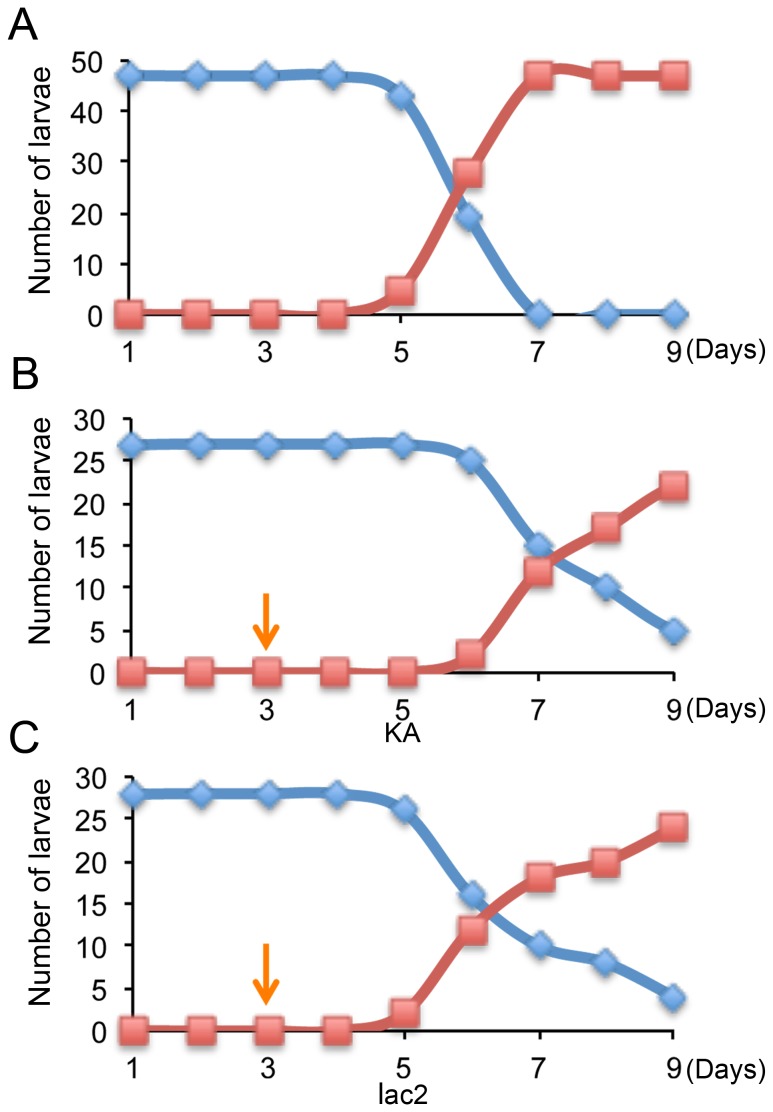
The effect of dsRNA feeding on the WCR life cycle. (A) The timing of the first larval molt with the artificial diet without dsRNA feeding. (B, C) The effect of dsRNA feeding on the timing of the first larval molt. (B) KA dsRNA fed, and (C) *lac2* dsRNA fed. Blue and red indicates the number of the first and second instar larvae, respectively. The orange arrow in B and C indicates the first day of dsRNA feeding.

To design a proper feeding schedule for the assay system, we first analyzed the life cycle of WCR. The majority of larvae molted into the second larval instar in 5 to 8 days after hatching (DAH) in the artificial diet system (Figure 5A). This information is critical, as body pigmentation phenotypes such as the *lac2* and *ebony* RNAi phenotypes can be observed only after a larval molt.

**Figure 6:**
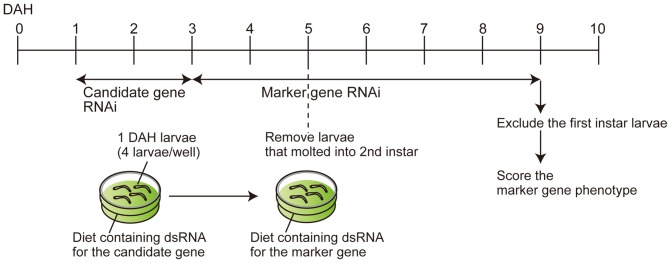
A two-step *in vivo* assay system for RNAi genes in WCR.

We also monitored the larval life cycle when larvae were fed with KA or *lac2* dsRNA (starting at 2 DAH) (Figure 5 B–C). dsRNA feeding slightly delayed larval growth, however, some larvae still molted around 5 DAH, and the majority of WCR molted by 8 DAH. In addition, our qPCR analysis showed that our feeding RNAi induces ∼90% reduction of mRNA levels in two days (although the efficiency does vary among genes) (Figure S3). Combining these findings, we opted for the following schedule for our assay system (Figure 6).

1. 1DAH: Start the first RNAi (for a candidate gene).
2. 3DAH: Start the second RNAi (marker gene RNAi).
3. 5DAH: Remove larvae that molted into the second instar. This will exclude the larvae that were fed with the marker dsRNA less than 2 days before molt.
4. 9DAH: Exclude the larvae that remained in the first instar, and score the marker gene RNAi phenotype.

### Establishing the *in vivo* assay system

Competition could occur among multiple RNAi treatments when two or more genes are targeted at the same time, which causes a reduction in the efficiency of individual RNAi (see [37] for example). This type of RNAi competition could complicate the WCR assay system, as the assay system relies on the interaction between two RNAi treatments. Both differences in length and dose of dsRNA between the first and second RNAi can significantly affect the degree of RNAi competition in the assay system.

We first tested the effect of the difference in dsRNA length between the first and second RNAi on the outcome of the assay system. Four different lengths (1000 bp, 750 bp, 500 bp, and 250 bp) of the negative control dsRNA (KA dsRNA) were used for the first RNAi at 10 µg/mL. 500 bp of *lac2* dsRNA was utilized for the second RNAi (with the same dose of 10 µg/mL). We also tested two different conditions for the second RNAi; (i) continuing the first RNAi throughout the assay period (*i.e.* the second step will be the double RNAi of the candidate and marker gene (co-feeding)), and (ii) discontinue the first RNAi once the second RNAi starts (sequential feeding). The co-feeding treatment can potentially increase the efficiency of the first RNAi, therefore, may make the assay system more sensitive to the knock down of the candidate genes. Hypothetically, none of these treatments would result in the suppression of the *lac2* RNAi (if there is no competition between two RNAi treatments), as the KA dsRNA molecules used in the first RNAi do not target any endogenous WCR gene. However, we saw significant suppression of the *lac2* RNAi phenotype by KA dsRNA feeding in the first RNAi treatment (up to 50% suppression; Table 1). This suppression appears to be dependent on the dsRNA length, with a longer dsRNA in the first RNAi being more efficient at competing with the second RNAi (Table 1). We also noticed that the co-feeding treatment induces more suppression of the second RNAi than sequential feeding (Table 1). These results indicate that it would be ideal to use the same length of dsRNA for both the first and second RNAi in the assay system, and that co-feeding may make the assay system overly sensitive to the presence of dsRNA molecules, causing the outcome to be less specific. Because the lengths of the fragments of some genes we cloned are shorter than 300 bp, we decided to adjust all dsRNA molecules used in the assay system to 250 bp.

**Table 1:** Evaluation of the assay system 1: dsRNA length-dependent competition.

1st RNAi	2nd RNAi		Initial # of larvae	# survived	# of 2nd instar	Lac2	WT	Suppression
1000 bp KA (10 ug/mL)	500 bp Lac2 (10 ug/mL)	co-feeding	20	15	15	7	8	53%
750 bp KA (10 ug/mL)	500 bp Lac2 (10 ug/mL)	co-feeding	19	16	13	8	5	38%
500 bp KA (10 ug/mL)	500 bp Lac2 (10 ug/mL)	co-feeding	18	17	10	6	4	40%
250 bp KA (10 ug/mL)	500 bp Lac2 (10 ug/mL)	co-feeding	16	15	11	7	4	36%

1st RNAi	2nd RNAi		Initial # of larvae	# survived	# of 2nd instar	Lac2	WT	Suppression
1000 bp KA (10 ug/mL)	500 bp Lac2 (500 ng/mL)	sequential	20	19	19	8	9	47%
750 bp KA (10 ug/mL)	500 bp Lac2 (500 ng/mL)	sequential	20	13	13	10	3	23%
500 bp KA (10 ug/mL)	500 bp Lac2 (500 ng/mL)	sequential	20	11	11	9	2	18%
250 bp KA (10 ug/mL)	500 bp Lac2 (500 ng/mL)	sequential	20	14	14	10	4	28%

The length of the dsRNA used in the initial RNAi affects the efficiency of the subsequent second RNAi. Various lengths of KA dsRNA were used for the initial RNAi (1st RNAi), while 500 bp *lac2* dsRNA was used for the second “marker gene” RNAi (2nd RNAi). The initial number of the larvae, the number of the larvae that survived the assay, and the number of larvae that became the second instar are indicated in the table. The second instar larvae were analyzed for their phenotypes, and categorized into the Lac2 phenotype (*i.e.* the second RNAi worked) and the WT phenotype (*i.e.* the second RNAi was suppressed). The suppression of the second RNAi phenotype by the initial RNAi was evaluated as the proportion of the WT larvae in the survived second instar larvae (Suppression).

### Validation of the assay system with positive controls

We next tested whether the RNAi for the core RNAi components (*Dcr2* and *Ago2*) can efficiently suppress the *lac2* RNAi (*i.e.* positive controls to test the sensitivity of the assay system). We utilized 250 bp dsRNA for *Dcr2* and *Ago2* for the first RNAi, and 250 bp *lac2* dsRNA for the second RNAi at 5 µg/mL for both the first and second RNAi treatments. To our disappointment, the negative control (KA dsRNA) suppressed the second RNAi as efficiently as RNAi for *Dcr2* or *Ago2*, either with the co-feeding or sequential feeding treatment (Table 2). We reduced the dose of the second RNAi (*lac2* RNAi) to 500 ng/mL to test whether we could make the assay system more specific to the genes involved in RNAi, however, the negative control still suppressed the second RNAi as well as RNAi for *Dcr2* and *Ago2* even in this condition (either co-feeding or sequential feeding) (Table 2). Together, these results indicate that *lac2* RNAi appears to be too sensitive to the presence of other dsRNA molecules, causing false positives in the assay system.

**Table 2:** Evaluation of the assay system 2: *lac2* as the marker gene.

1st RNAi	2nd RNAi		Initial # of larvae	# survived	# of 2nd instar	lac2	WT	suppression
250 bpAgo2 (5 ug/mL)	250 bp lac2 (5 ug/mL)	co-feeding	30	26	19	18	1	5%
250 bp Dcr2 (5 ug/mL)	250 bp lac2 (5 ug/mL)	co-feeding	28	21	21	17	4	19%
250 bp KA (5 ug/mL)	250 bp lac2 (5 ug/mL)	co-feeding	27	24	24	17	7	29%
250 bpAgo2 (5 ug/mL)	250 bp lac2 (5 ug/mL)	sequential	30	22	16	15	1	6%
250 bpDcr2 (5 ug/mL)	250 bp lac2 (5 ug/mL)	sequential	28	13	12	12	0	0%
250 bp KA (5 ug/mL)	250 bp lac2 (5 ug/mL)	sequential	27	20	15	11	4	26%

1st RNAi	2nd RNAi		Initial # of larvae	# survived	# of 2nd instar	lac2	WT	suppression
250 bpAgo2 (5 ug/mL)	250 bp lac2 (500 ng/mL)	co-feeding	13	9	9	6	3	33%
250 bpDcr2 (5 ug/mL)	250 bp lac2 (500 ng/mL)	co-feeding	11	9	7	6	1	14%
250 bp KA (5 ug/mL)	250 bp lac2 (500 ng/mL)	co-feeding	10	8	8	7	1	12%
250 bpAgo2 (5 ug/mL)	250 bp lac2 (500 ng/mL)	sequential	30	21	20	18	2	10%
250 bpDcr2 (5 ug/mL)	250 bp lac2 (500 ng/mL)	sequential	28	20	18	17	1	5%
250 bp KA (5 ug/mL)	250 bp lac2 (500 ng/mL)	sequential	27	20	20	16	4	20%

The amount of the maker gene dsRNA and the feeding scheme influence the outcome of the assay. The second instar larvae were categorized into the Lac2 phenotype (*i.e.* the second RNAi worked) and the WT phenotype (*i.e.* the second RNAi is suppressed). The suppression of the second RNAi phenotype by the initial RNAi was evaluated as the proportion of the WT larvae in the survived second instar larvae (Suppression).

**Figure 7:**
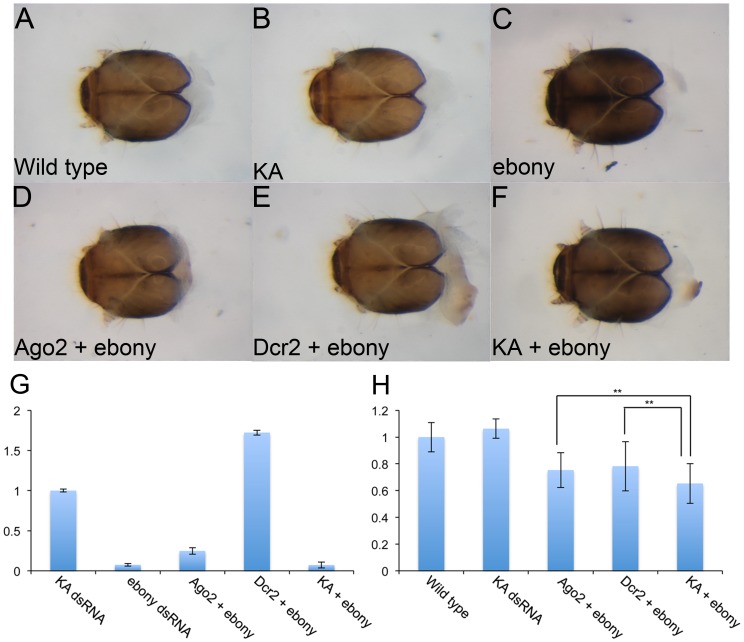
RNAi for *Dcr2* and *Ago2* efficiently suppress the marker gene RNAi. (A–F) The head capsule of wild-type (A), KA dsRNA (B), *ebony* RNAi (C), *Ago2* + *ebony* RNAi (D), *Dcr2* + *ebony* RNAi (E), and KA + *ebony* RNAi (F). Both *Ago2* and *Dcr2* RNAi, but not KA dsRNA, suppress the *ebony* RNAi phenotype (D, E). (G) qPCR quantification of *ebony* mRNA. The Y-axis indicates relative expression levels compared to the control (KA dsRNA). (H) ImageJ analysis for the *Ago2* and *Dcr2* assay results. The Y-axis indicates the pigmentation index with 1 being the wild-type mean gray value. Both *Ago2* + *ebony* RNAi and *Dcr2* + *ebony* RNAi larvae have significantly higher pigmentation values than the KA + *ebony* RNAi control (P**≤0.01. n = 40 for KA and n = 20 for other experiments).

Although we could potentially further change the parameters for *lac2* RNAi to identify the optimal condition of *lac2* RNAi for the assay system, instead we decided to try the other marker gene that we have identified, *ebony*, for the assay system. We first tested the same dose and the same length of dsRNA for the first and second RNAi (250 bp, 5 µg/mL). We evaluated these conditions with either the co-feeding or sequential feeding treatment. The negative control treatment still suppressed the second (*ebony*) RNAi as efficient as the positive controls with co-feeding (Table 3). In contrast, the second RNAi was specifically suppressed by the positive controls (*Ago2* and *Dcr2* RNAi), but not by the negative control (KA dsRNA) with the sequential feeding treatment (Table 3). As the suppression efficiency of the second RNAi by the positive controls was relatively low (18% by *Ago2* RNAi and 5% by *Dcr2* RNAi), we determined if reducing the dose of dsRNA for the second RNAi would make the suppression more visible (*i.e.* making the assay system more sensitive). We used 500 ng/mL of *ebony* dsRNA for the second RNAi with either co-feeding or sequential feeding. While the co-feeding treatment induced even more non-specific suppression of the second RNAi (up to 60%) (Table 3), the sequential feeding treatment with this *ebony* dsRNA dose induced stronger suppression of the second RNAi by the positive controls (55% by *Ago2* or *Dcr2* RNAi) than by the negative control (23% with KA dsRNA) (Table 3 and Figure 7 A–F). The positive control specific suppression was also confirmed by qPCR as well as by the ImageJ analysis (Figure 7 G–H). Although we could potentially improve the assay system further by changing the dsRNA length and dose (also see Discussion regarding a potential caveat of this “RNAi on RNAi” treatment), this condition appears to be sufficient to differentiate positive outcomes from non-specific suppression of the second RNAi.

**Table 3:** Evaluation of the assay system 3: *ebony* as the marker gene.

1st RNAi	2nd RNAi		Initial # of larvae	# survived	# of 2nd instar	ebony	WT	suppression
250 bp Ago2 (5 ug/mL)	250 bp ebony (5 ug/mL)	co-feeding	30	24	18	13	5	27%
250 bp Dcr2 (5 ug/mL)	250 bp ebony (5 ug/mL)	co-feeding	28	26	25	19	6	24%
250 bp KA (5 ug/mL)	250 bp ebony (5 ug/mL)	co-feeding	27	24	19	13	6	31%
250 bp Ago2 (5 ug/mL)	250 bp ebony (5 ug/mL)	sequential	30	20	16	13	3	18%
250 bp Dcr2 (5 ugmL)	250 bp ebony (5 ug/mL)	sequential	28	21	20	19	1	5%
250 bp KA (5 ug/mL)	250 bp ebony (5 ug/mL)	sequential	27	14	13	13	0	0%

1st RNAi	2nd RNAi		Initial # of larvae	# survived	# of 2nd instar	ebony	WT	suppression
250 bp Ago2 (5 ug/mL)	250 bp ebony (500 ng/mL)	co-feeding	10	10	8	4	4	50%
250 bp Dcr2 (5 ug/mL)	250 bp ebony (500 ng/mL)	co-feeding	8	6	5	2	3	60%
250 bp KA (5 ug/mL)	250 bp ebony (500 ng/mL)	co-feeding	7	6	5	2	3	60%
250 bp Ago2 (5 ug/mL)	250 bp ebony (500 ng/mL)	sequential	30	21	20	9	11	55%
250 bp Dcr2 (5 ug/mL)	250 bp ebony (500 ng/mL)	sequential	28	21	18	8	10	55%
250 bp KA (5 ug/mL)	250 bp ebony (500 ng/mL)	sequential	27	22	17	13	4	23%

Evaluation of the assay system with *ebony* as the marker gene instead of *lac2*. The second instar larvae were categorized into the *ebony* phenotype (*i.e.* the second RNAi worked) and the WT phenotype (i.e. the second RNAi is suppressed). The suppression of the second RNAi phenotype by the initial RNAi was evaluated as the proportion of the WT larvae in the survived second instar larvae (Suppression).

Taken together, these results indicate that *ebony* can be used as a marker gene for the assay system, and “First RNAi: 250 bp, 5 µg/mL + Second RNAi: *ebony* dsRNA 250 bp, 500 ng/mL, sequential feeding” with the schedule described in the previous section would work specifically enough to evaluate the involvement of genes of interest in WCR RNAi.

### Potential involvement of *sid-1-like* genes in WCR environmental RNAi

*sid-1* encodes a dsRNA channel protein, which is critical for the systemic RNAi response in *C. elegans*. Many insects also have genes similar to *sid-1* (*sid-1-like* gene, *sil*) [11], however, the involvement of the *sil* genes in systemic RNAi in insects is still largely unknown. We decided to utilize the assay system established in this study to assess the involvement of these *sil* genes in environmental RNAi in WCR.

**Figure 8:**
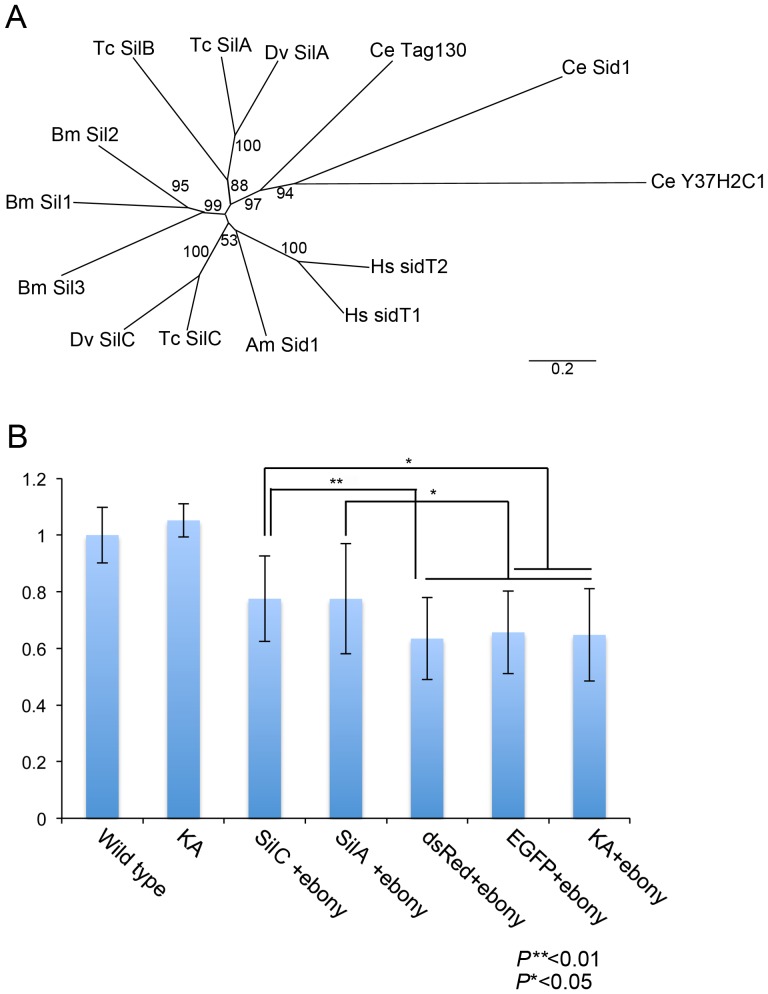
Potential involvement of *sil* genes in WCR environmental RNAi. (A) Phylogenetic analysis for Sil proteins. (B) ImageJ analysis for the Dv-*silA* and *silC* assay results. The Y-axis indicates the pigmentation index with 1 being the wild-type mean gray value. Both Dv-*silA* + *ebony* RNAi and Dv-*silC* + *ebony* RNAi larvae have significantly higher pigmentation values than the controls (KA, *dsRed*, or *EGFP* + *ebony* RNAi) (P**<0.01 or P*<0.05. n = 40 for KA and n = 20 for other genes).

We identified two *sil* genes from the WCR unigene database, each of which is orthologous to *Tc-silA* and *Tc-silC*, respectively (Figure 8 A). We evaluated these two genes by our assay system. Both Dv-*silA* and Dv-*silC* RNAi showed greater than 50% knockdown efficiency of their respective mRNA levels (Figure S3). In addition, we added two more negative control experiments (*dsRed* and *EGFP*) to make sure that our assay system specifically responds to the genes involved in RNAi. The results for two WCR *sil* genes were positive (block RNAi of second dsRNA of marker gene) while none of the mock dsRNA treatments were positive (Figure 8 B), suggesting that both *Dv-silA* and *Dv-silC* are involved in WCR environmental RNAi. However, the suppression of the *ebony* RNAi phenotype by RNAi for *sil* genes was not strong, which may suggest that *sil* genes are involved only in a part of the WCR environmental RNAi processes (see Discussion for details). Further analysis will be required to determine the precise involvement of the *sil* genes in environmental RNAi.

## Discussion

In this study, we have established an *in vivo* assay system in WCR that will allow a quick survey of the genes that may be involved in environmental RNAi in WCR. We have identified two pigmentation genes, *lac2* and *ebony*, as marker genes for our assay system, and implemented ImageJ to quantify pigmentation defects caused by RNAi for these marker genes. Although RNAi for either *lac2* or *ebony* produced a scoreable pigmentation phenotype, the *ebony* RNAi phenotype appears to be more stable in the presence of additional dsRNA molecules, making *ebony* a more suitable marker gene for our assay system. We have also identified two core RNAi genes, *Ago2* and *Dcr2*, from the WCR unigene dataset, and used them as positive controls to test our assay system. After several adjustments to the dsRNA length and dose, as well as to the feeding schedule, we were able to establish an assay system that specifically responds to positive controls.

### dsRNA length-dependent competition

Having a mixture of dsRNA often results in competition between the dsRNAs for the RNAi components, including the core RNAi machinery as well as the cellular uptake/transport components (for example, see [36], [37]). These competitions are usually dose-dependent, in which an RNAi treatment with more dsRNA molecules wins out the other RNAi treatment [36], [37]. In the process of establishing the assay system, we noticed that, in WCR, competition occurs not only depending on the amount of the dsRNA molecules fed, but also depending on their lengths. Longer dsRNA molecules outcompeted shorter dsRNA molecules in our WCR feeding RNAi experiments (Table 1). Two lines of observation suggest that this length-dependent competition occurs at the cellular dsRNA uptake/spreading level; (i) in many organisms, dsRNA, once delivered into the inside of the cell, can trigger an efficient RNAi response regardless of its length (see [37] for example), (ii) the efficiency of cellular dsRNA uptake depends on the length of dsRNA in WCR [25], in *Tribolium* [37], and in *C. elegans* [16]. It would be interesting to analyze if the length-dependent competition is specific to intestinal cells, or universal to most cells in WCR. This can be analyzed by comparing feeding RNAi with dsRNA injection in WCR. Evaluating the presence of the length-dependent competition in *Tribolium* might also be informative, as *Tribolium* lacks a robust environmental RNAi response despite the presence of strong systemic RNAi (data not shown). Elucidating tissue and species specificity of the length-dependent RNAi competition may give us a clue to understand the molecular basis of environmental RNAi.

### RNAi on RNAi

In this study, we utilized two core RNAi genes, *Ago2* and *Dcr2*, as the positive controls for our assay system. Although we detected significant suppression of marker gene knockdown by *Ago2* and *Dcr2* RNAi, the suppression was not strong (20–30% more suppression compared to the negative control, Table 3). We have previously seen a similar tendency in *Tribolium* [11]. This lack of robust suppression might be due to the “RNAi on RNAi” nature of this experiment. RNAi for a gene involved in RNAi itself (such as *Ago2* and *Dcr2*) will initially induce the suppression of RNAi. However, this suppression will prevent further suppression of RNAi due to the lack of RNAi machinery, leading to de-suppression of RNAi. This de-suppression in turn will allow cells to regain RNAi machinery, causing the second wave of RNAi suppression. Therefore, in theory, RNAi efficiency in the RNAi-on-RNAi individual should oscillate, which may account for the lack of robust suppression in our positive control experiments. It is yet to be determined whether RNAi for genes involved in environmental RNAi will cause this type of complex RNAi oscillation. Since RNAi effect can persist well after providing dsRNA molecules [37], it is possible that the genes important for the environmental/systemic aspect of RNAi might not be essential once RNAi is initiated. If this is the case, RNAi for genes involved in environmental RNAi would not affect the function and efficiency of RNAi machinery, therefore would result in much more robust suppression of the marker gene RNAi in our assay system.

### Sid-1 like genes: essential or dispensable in insect systemic RNAi?

Sid-1 dsRNA channel is an indispensable component for systemic RNAi in *C. elegans*. Although insects possess a gene similar to *sid-1* (*sid-1-like* gene, *sil*) [11], whether these genes play roles in insect systemic RNA is largely unknown. Since *Drosophila*, which lacks *sil* genes, also lacks a robust systemic RNAi response, the correlation between the presence of a robust systemic RNAi response and the presence of the *sid-1* homologs was proposed. However, this correlation has been challenged. For example, mosquito species do not possess *sil* genes, yet they exhibit a systemic RNAi response. On the other hand, lepidopteran insects have multiple *sil* genes, yet are very poor at exhibiting systemic RNAi. Recently, the ability of a lepidopteran cultured cell-line to take up dsRNA molecules from cultured media was tested, demonstrating that lepidopteran cells are poor at taking up dsRNA from the media even though the lepidopteran *sil* genes are expressed in these cells [39], [40]. Interestingly, overexpression of *C. elegans sid-1* in these cells dramatically enhance the ability of the lepidopteran cell to take up dsRNA from the culture media [39], [40], suggesting the functional differences between insect *sil* and *C. elegans sid-1*. Furthermore, a detailed amino acid sequence comparison between insect and nematode Sid-1 homologs has revealed that insect *sil* are more similar to another *C. elegans* gene, *tag-130*, which is dispensable from systemic RNAi in *C. elegans* [11]. There have been several reports both supporting and opposing the function of insect *sil* genes in systemic RNAi (see [12] for review). Therefore, the involvement of insect *sil* genes in systemic and environmental RNAi is yet to be determined.

By utilizing our assay system, we have evaluated the involvement of *sil* genes in environmental RNAi in WCR. The results were positive for both *sil* genes, suggesting that the *sil* genes are indeed involved in WCR environmental RNAi. However, the suppression of the marker gene RNAi by *sil* RNAi was not robust, which may indicate that the *sil* genes are involved in the environmental RNAi processes only partially. Alternatively, it is also possible to think that the two *sil* genes act redundantly in the environmental RNAi processes, rescuing the marker gene RNAi suppression. We have attempted *sil A*+*C* double RNAi, however, we had limited success adjusting our assay system for the double RNAi condition. Further modifications to the assay system are required to be able to assess the possibility of functional redundancy between the two *sil* genes in WCR.

We have previously assessed the involvement of *sil* genes in systemic RNAi (via larval injection) by using a similar assay system in *Tribolium* [11]. *Tribolum* has three *sil* like genes (Figure 8 A), but none of each single RNAi or triple RNAi interfered with the subsequent marker gene RNAi [11]. There is a caveat to this result, as these *sil* genes in *Tribolium* could be acting redundantly in each single RNAi, while triple RNAi could potentially trigger RNAi competition, lowering the efficiency of *sil* RNAi itself. Nonetheless, it is interesting that the results came out differently between these two beetles. One striking difference between WCR and *Tribolium* is that WCR shows a robust systemic environmental RNAi response while *Tribolium* does not (data not shown) (but also see [41] for the presence of a environmental RNAi response in *Tribolium*). Therefore, it is intriguing to speculate that the *sil* genes are predominantly involved in the intestinal dsRNA uptake and/or dsRNA spreading from intestine in WCR. Evaluating dsRNA uptake efficiency in the intestinal cells by using an *in vitro* culture system in combination with *sil* RNAi may give us more insights into the functions of the *sil* genes in WCR.

### Candidate genes and genome-wide survey

The next step is to utilize the assay system and evaluate more candidate genes whose orthologs have been implicated in systemic RNAi in other organisms. As mentioned, the molecular basis of systemic RNAi has been studied most extensively in *C. elegans*, in which a battery of critical systemic RNAi genes has been identified. In addition to *sid-1* mentioned above, three genes, *rsd-2*, *rsd-3*, and *rsd-6*, have been identified to be important for the germ-line related systemic RNAi response [42]. *sid-2* is another essential systemic RNAi gene in *C. elegans* [43]. *sid-2* codes for a transmembrane protein, and is critical specifically for the intestinal dsRNA uptake step [43]. More recently, two more genes, *sid-3* and *sid-5*, have also been identified to be important for systemic RNAi in *C. elegans* [17], [44]. Although some of these genes are unique in *C. elegans* (e.g. *sid-2*), others have orthologs in WCR. It would be interesting to test the involvement of these genes in the WCR environmental RNAi.

Another pool of candidate genes comes from *Drosophila* S2 cell studies [19], [20]. These studies have identified over 20 genes that are potentially involved in the dsRNA uptake process [19], [20]. Many of them are implicated in endocytosis, suggesting the presence of an endocytosis-based dsRNA uptake mechanism in insects. Most of these genes have beetle orthologs [11], and are therefore good candidates to be evaluated by our assay system.

Our assay system may also be adaptable to a genome-wide high-throughput RNAi screening. High-throughput RNAi screenings have been quite successful in *C. elegans*, where dsRNA can be easily supplied via feeding (reviewed in [45]). In addition, the *Drosophila* cultured cell system has also been utilized for high-throughput RNAi screenings for genes involved in various cellular processes [46], [47]. However, adapting a high-throughput RNAi screening to other organisms has been a challenge, because of the difficulty of delivering dsRNA molecules into the organisms. The ease of feeding RNAi in WCR may allow us to perform a high-throughput screening *in vivo*, which leads to the identification of genes involved in environmental RNAi without depending on previously identified candidate genes.

Surveying various sets of genes in WCR with the assay system established in this study, followed by functional analyses for the genes identified through the assay, allows us to approach the molecular basis of WCR environmental RNAi. Detailed knowledge of the molecules and mechanisms responsible for environmental RNAi will help determine an efficient way of utilizing RNAi for insect pest management.

## Methods

### Insects

For all bioassays, WCR eggs were received from Crop Characteristics (Farmington, MN). Eggs were maintained at a target temperature of 10°C to 25°C depending on desired hatch time prior to disinfection. Eggs and WCR diet plates were shipped from Monsanto Research facility. Near-hatching eggs were washed and dispensed into plastic containers prior to hatching. Newly hatched neonates (<30 hours post hatch) were used in all assays. WCR artificial diet [25] was used for feeding bioassays with dsRNA.

### Gene Identification and Phylogenetic Analysis

WCR in-house transcriptome 454 reads and ESTs from NCBI were assembled into unigene using Newbler with default settings. The unigene contig sequences were then translated into corresponding peptide sequences based on sequence similarity comparisons against non-redundant peptide dataset uniRef90 [48]. Any detected sequencing errors in unigene contigs were corrected during translation. The translated dataset was used for homologous gene identification and phylogenetic analysis to minimize potential noises introduced by transcriptome sequencing errors.

For homologous gene identification, reciprocal best blast hits were used between WCR unigene and *Tribolium castaneum* genome peptide set. Then pfam domains [49] were searched to identify query genes' hallmark domains in WCR candidate peptide sequences as a way to validate the reciprocal blast approach. To build phylogenetic trees, we used MEGA program package [50], in which multiple sequence alignment was performed with ClustalW algorithm and trees were constructed using Neighbor-joining algorithm with bootstrap of 1000 replications.

For *yellow* genes, *Tribolium* Yellow protein sequences [32] were used as queries. After WCR Yellow proteins were identified, the signature pfam domain of “MRJP” was located from each Yellow protein by pfam hmmsearch program. This domain was used for multiple sequence alignment and phylogenetic tree construction. WCR Ago2 and Dcr2 were identified from WCR unigene datase by using *C. elegans* and *Tribolium* Ago and Dcr proteins as queries. Trees for Ago and Dcr were based on Piwi domain and pfam domain “Ribonuclease_3”, respectively. WCR Sil sequences were identified by using *Tribolium* Sil [11] as queries. Nine short WCR contigs were aligned to different regions of *Tribolium* Sil proteins. RT-PCR experiment was carried out to stitch the short sequences, resulting in two longer sequences which are designated as “DvSi1A” and “DvSilC” in this study. Pfam domain of “SID-1_RNA_chan” was used for phylogenetic analysis.

### Gene Cloning

Total RNA was isolated from WCR second instar larvae by Maxwell 16 LEV simlyRNA tissue Kit (Promega), and cDNA was synthesized with SuperScript III (Invitrogen) using oligo dT primer. The cDNA fragments of the genes of interest were then amplified by PCR, and cloned into pCR4-TOPO using the TOPO TA Cloning Kit for Sequencing (Invitrogen). The cloning primers and sequences for the WCR genes identified in this study are included in Table S2 and Document S1, respectively.

### dsRNA Synthesis

The dsRNA templates were synthesized by PCR using the TOPO_RNAi primer or gene specific primers with the T7 polymerase promoter sequence at the 5′ end. The primer sets and their sequences used to produce these dsRNA templates are in Table S1. For the 30 bp dsRNA molecules used in this study, we used de novo synthesized oligos to produce the dsRNA templates (Table S1). The sense and anti-sense oligos including 30 bp of the *lac2* or *ebony* coding region with a T7 promoter sequence at their 5′ ends were annealed to produce the double- stranded DNA template for dsRNA synthesis (50°C for 20 minutes for annealing). dsRNAs were synthesized by *in vitro* transcription (Megascript T7, Ambion) and then purified by MegaClear kit (Ambion) as described before, except for the dsRNA molecules shorter than 100 bp. Since MegaClear kit removes RNA molecules shorter than 100 bp, we did conventional phenol/chloroform extraction followed by ethanol precipitation for the purification of the 50 bp and 30 bp dsRNA molecules.

### dsRNA Feeding

10 µl of dsRNA solution with an appropriate concentration was added to each well of a 96-well diet plate (200 µl diet/well), and air-dried. Four first larval instar larvae were placed per well for dsRNA feeding treatment. The plate was sealed with transparent Scotch tape with a ventilation hole on each well, and incubated at 25°C with 70% humidity. For all negative control treatments, dsRNA produced from a part of Kanamycin and Ampicillin resistance genes in the pCR4-TOPO vector (KA dsRNA) has been used. Feeding KA dsRNA does not affect larval pigmentation or larval mortality (Figure 1 D–F).

### Real-time RT-PCR

RNA was extracted from WCR second larval stage larvae by Maxwell 16 LEV simlyRNA tissue Kit (Promega), and cDNA was synthesized by iScript cDNA Synthesis Kit (Bio-Rad). Real-time PCR was performed by using SsoAdvanced SYBR Green Supermix with CFX Connect Real-Time PCR Detection System (Bio-Rad). *tubulin* and *GAPDH* were used as the two reference genes for ΔΔCq quantification. The amplification efficiency for all qPCR primer sets was tested prior to quantification. qPCR primer sets and their amplification efficiency are in Table S3.

### Image J analysis

ImageJ 1.47 [38] was used for quantifying the larval head pigmentation. Larval heads were dissected in 95% ethanol, and documented by Zeiss AxioCam MRc5 with Zeiss Discovery V12 (at 100X magnification and 2584 X 1936 pixels, with a constant light intensity). A 10 µm^2^ square in the middle of the left half of each larval head was used for the ImageJ analysis (Figure S4). The selected area was first converted to 8-bit gray scale, and then analyzed for the Mean Gray value. The Mean Gray value of each larva from the experimental groups was divided by the mean of the wild-type Mean Gray value to obtain the pigmentation index. For each experiment, 10–40 larvae were randomly chosen and analyzed. Statistic significance was determined by two-tailed t-test with unequal variance at P*<0.05, P**<0.01 and P***<0.001.

## Supporting Information

Figure S1 **Phylogenetic analysis of Yellow proteins.** *Dv-yellow-CLUS03632* (*yellow-f*) and *Dv-yellow-CLUS05743* (*yellow-c*) were analyzed in this study.
(TIF)

Figure S2 **Feeding RNAi for** ***yellow*** **genes in WCR.** (A–C) wild-type, (D–F) *yellow-c* RNAi, (G–I) *yellow-f* RNAi, and (J–L) *yellow-c* and *yellow-f* double RNAi. Neither each single nor double RNAi affected the larval pigmentation.
(TIF)

Figure S3 **Feeding RNAi efficiency in WCR.** (A–F) Reduction of mRNA by feeding RNAi for *yellow-c* (A), *yellow-f* (B), *lac2* (C), *ebony* (D), *Ago2* (E) *Dcr2* (F), Dv-*SilC* (G) and Dv-*SilA* (H). KA dsRNA was used as a negative control. mRNA levels were quantified by qPCR 2 days after the beginning of dsRNA feeding. Note that feeding RNAi causes 80–90% reduction of mRNA in WCR.
(TIF)

Figure S4 **Quantitative analysis for larval head pigmentation.** (A–C) the location of the 10 µm^2^ square in the head capsule of the KA dsRNA fed larva (A), *lac2* RNAi (B), and *ebony* RNAi (C). (D–E) Complied squares (D) and the gray scale converted squares (E). These gray scale squares were then analyzed by Image J to obtained the Mean Gray Value (F).
(TIF)

Table S1 **dsRNA used in this study.**
(XLSX)

Table S2 **Genes cloned in this study.**
(XLSX)

Table S3 **qPCR primers, amplicon length, and amplification efficiency.**
(XLSX)

Document S1 **Sequences of the genes identified in this study.** Both the nucleotide sequences of the genes and their translated amino acid sequences are included. In the case that a cloned cDNA fragment contains stop codons in the correct frame, the portion of the amino acid sequence that corresponds to the coding region is highlighted with red.
(DOCX)
