# Supplementary figures and images for: Establishing an In Vivo Assay System to Identify Components Involved in Environmental RNA Interference in the Western Corn Rootworm
Source: PLoS One. 2014 Jul 8;9(7):e101661. doi: 10.1371/journal.pone.0101661 (PMC4086966; doi:10.1371/journal.pone.0101661)

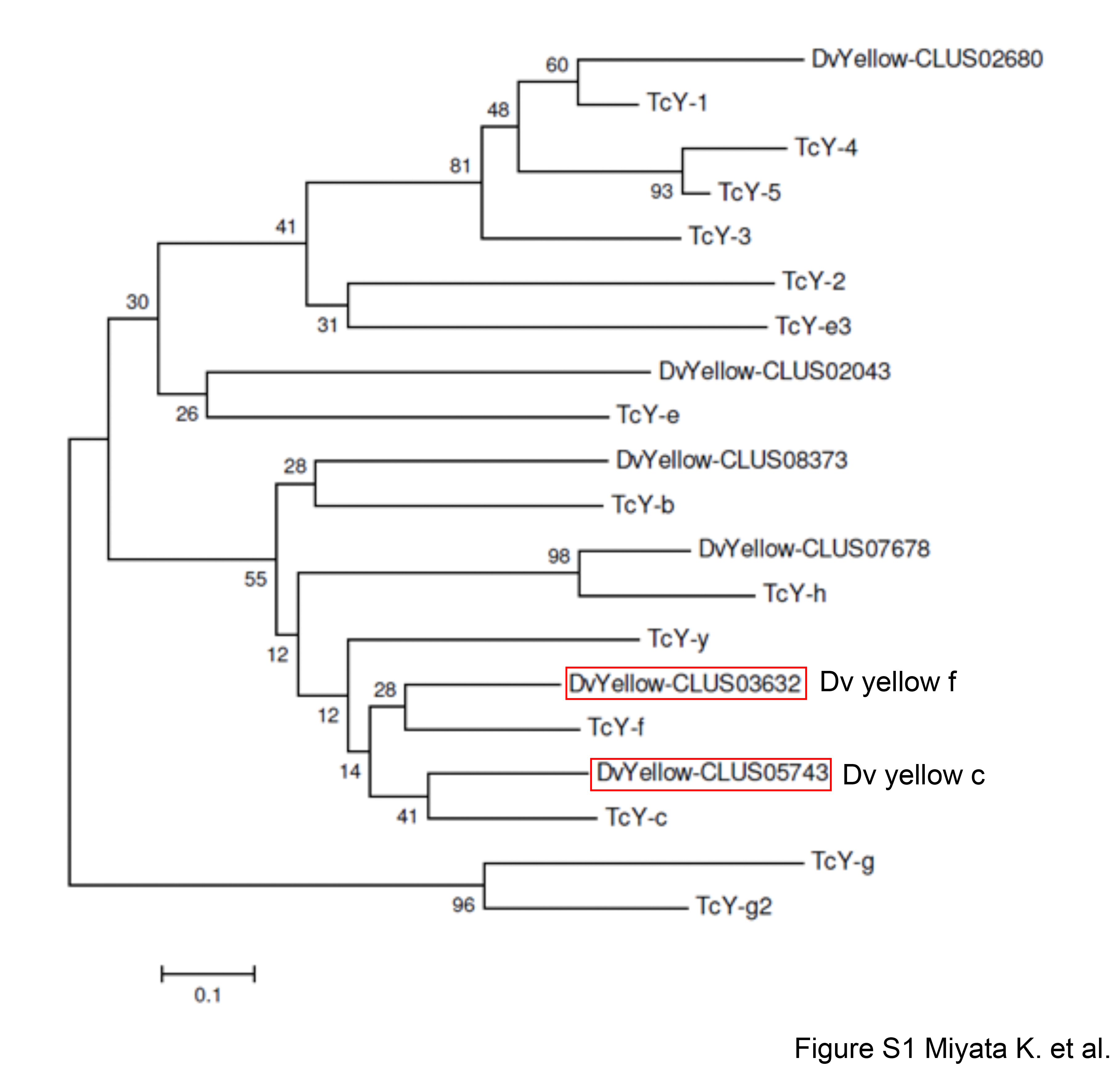

Supplement: Figure S1 — Phylogenetic analysis of Yellow proteins. Dv-yellow-CLUS03632 (yellow-f) and Dv-yellow-CLUS05743 (yellow-c) were analyzed in this study. (TIF) [file pone.0101661.s001.tif]

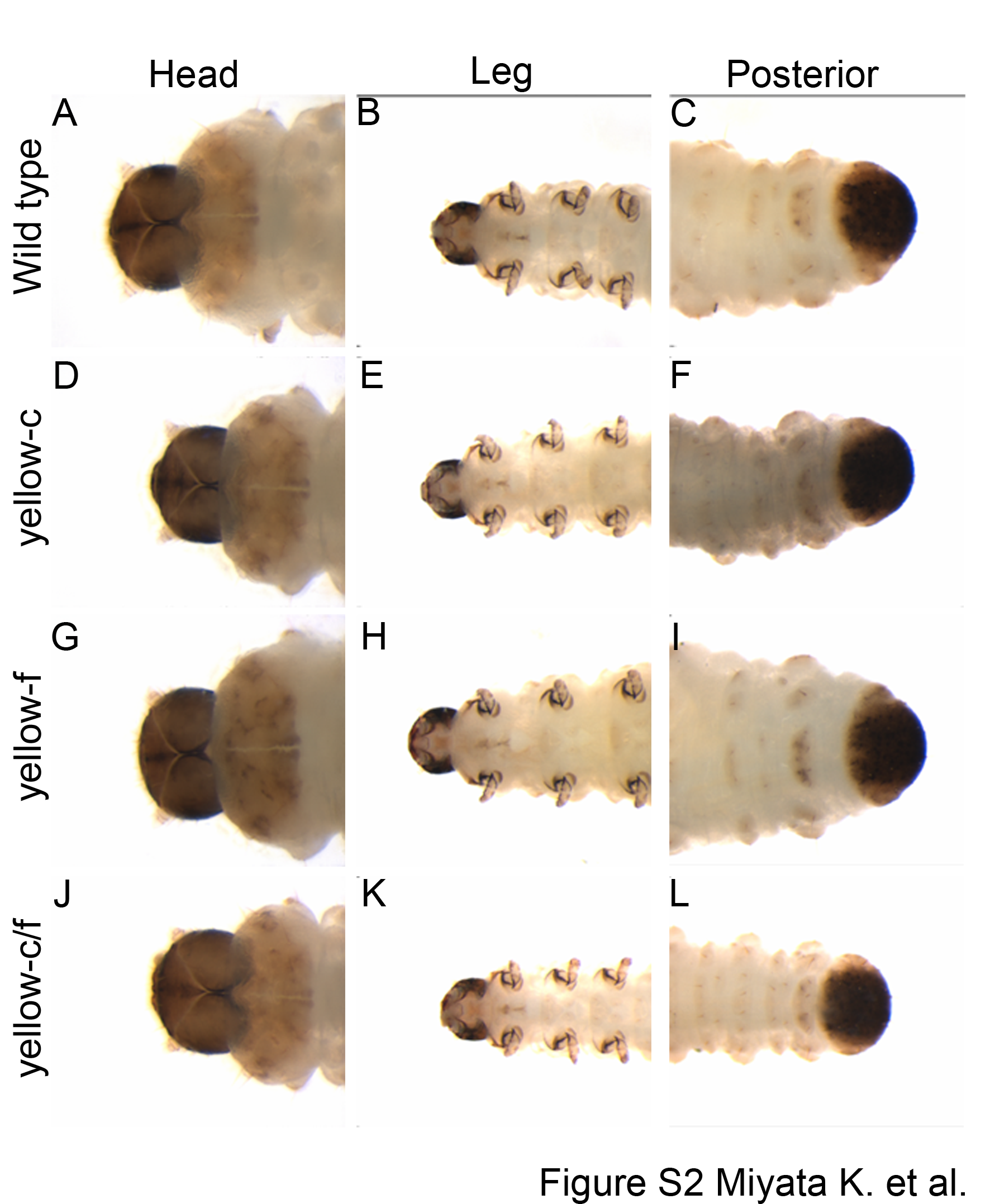

Supplement: Figure S2 — Feeding RNAi for yellow genes in WCR. (A–C) wild-type, (D–F) yellow-c RNAi, (G–I) yellow-f RNAi, and (J–L) yellow-c and yellow-f double RNAi. Neither each single nor double RNAi affected the larval pigmentation. (TIF) [file pone.0101661.s002.tif]

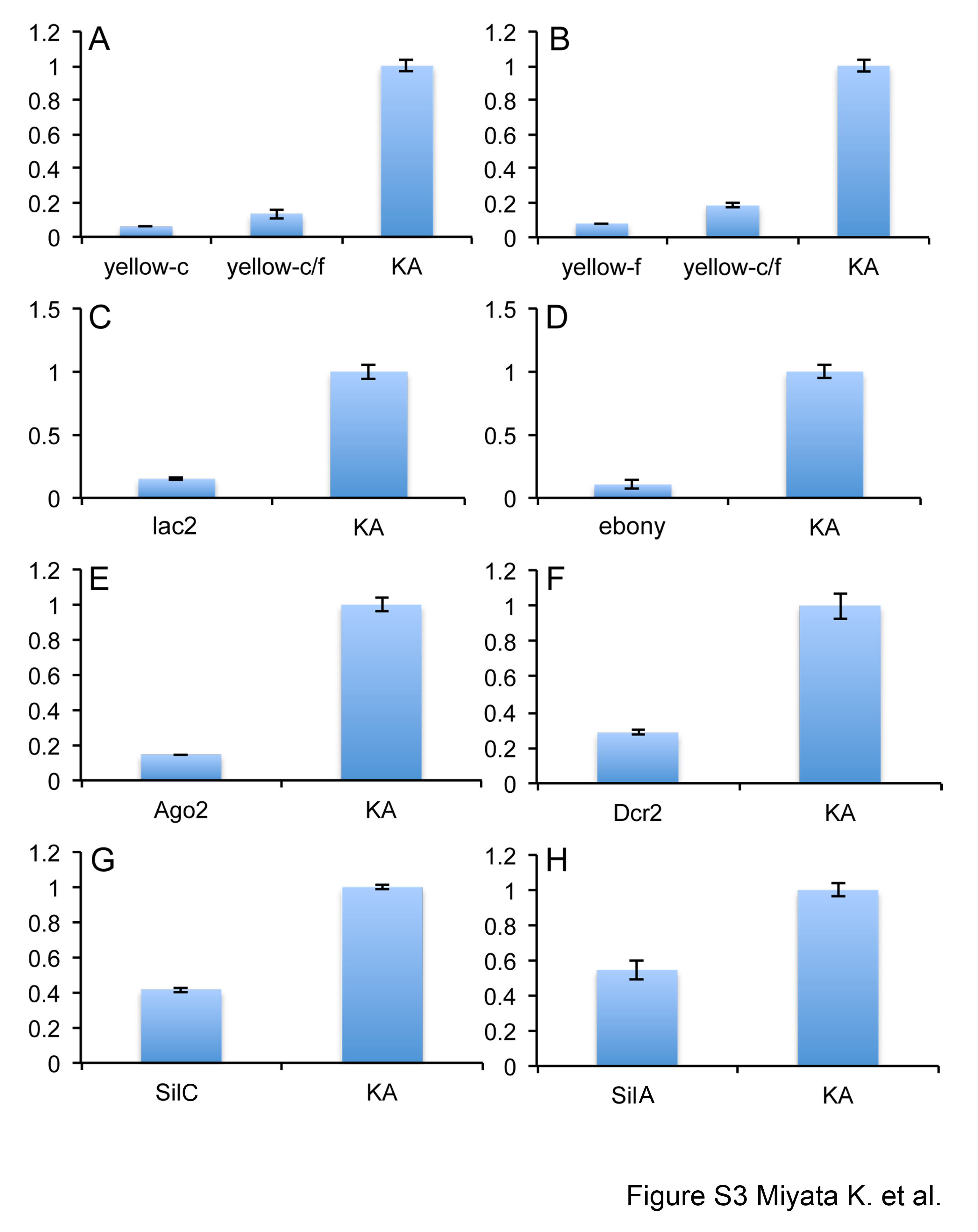

Supplement: Figure S3 — Feeding RNAi efficiency in WCR. (A–F) Reduction of mRNA by feeding RNAi for yellow-c (A), yellow-f (B), lac2 (C), ebony (D), Ago2 (E) Dcr2 (F), Dv-SilC (G) and Dv-SilA (H). KA dsRNA was used as a negative control. mRNA levels were quantified by qPCR 2 days after the beginning of dsRNA feeding. Note that feeding RNAi causes 80–90% reduction of mRNA in WCR. (TIF) [file pone.0101661.s003.tif]

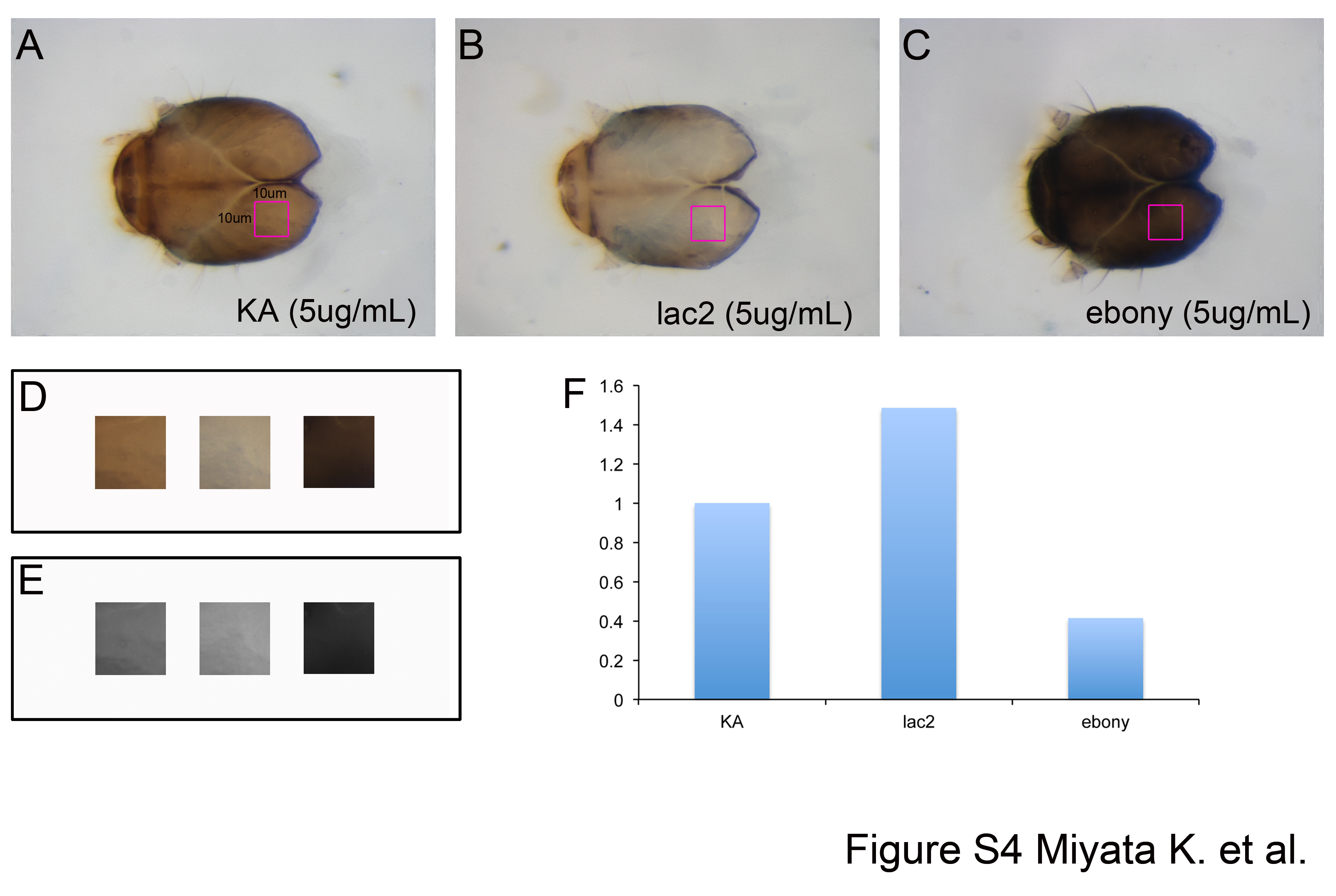

Supplement: Figure S4 — Quantitative analysis for larval head pigmentation. (A–C) the location of the 10 µm2 square in the head capsule of the KA dsRNA fed larva (A), lac2 RNAi (B), and ebony RNAi (C). (D–E) Complied squares (D) and the gray scale converted squares (E). These gray scale squares were then analyzed by Image J to obtained the Mean Gray Value (F). (TIF) [file pone.0101661.s004.tif]
