# Supplementary material for: Establishing an In Vivo Assay System to Identify Components Involved in Environmental RNA Interference in the Western Corn Rootworm
Source: PLoS One. 2014 Jul 8;9(7):e101661. doi: 10.1371/journal.pone.0101661 (PMC4086966; doi:10.1371/journal.pone.0101661)
Supplement: Document S1 — Sequences of the genes identified in this study. Both the nucleotide sequences of the genes and their translated amino acid sequences are included. In the case that a cloned cDNA fragment contains stop codons in the correct frame, the portion of the amino acid sequence that corresponds to the coding region is highlighted with red. (DOCX) [file pone.0101661.s008.docx]

**Document S1: Sequences of the genes identified in this study**

**>yellow-c**

aatttgaacgttggcggagtcaatttccagtggactgatggagtgtttagcattgctctaggaccaataaatgaaacaactggctacagaacagcttatttccatgctctagcaagttgcaatgaattttccgtcagcacggaagttttgaaaaatgaactactggcttcagatccatcctcgtttgatatgtacaaacttgaaggatttaagaatgaccaaggacaatctacatcttcggaattcgacgttaaagcaaatgtcttgttcacgactcaaatacaaagggatgctatcgcttgttggaatcctagaaaaaaactcgatgctaacacattcgcactggttgcacaggatcacgaaaaactgattttcactaatgatatctcgattgatgctgaaagaaacctctgggtgttatcggacagattgccagcatttatttacagagaactaaatccgaaccaaatcaa

473 bp

F1: aatttgaacgttggcggagtc

R1: ttgatttggttcggatttagttc

Frame+1

Amino acid

NLNVGGVNFQWTDGVFSIALGPINETTGYRTAYFHALASCNEFSVSTEVLKNELLASDPSSFDMYKLEGFKNDQGQSTSSEFDVKANVLFTTQIQRDAIACWNPRKKLDANTFALVAQDHEKLIFTNDISIDAERNLWVLSDRLPAFIYRELNPNQI

**>yellow-f**

acgctggggctgggggtagtagcagtcctataatctacgaggatcccaatttgcccaacagcggatggaatccttcagaaggaatcgattataatcatgttacgaacattcctatgggtgcaaacgtatggaggaacaagctcttcatcacggttcctagaagacgactaggagtgccatctacattgaactatgtgccattggacagtccaagaagacacaacgttccgctgattccctacccagattgggataccaatatatatccagatcccaaaaacagcggtgataattttgtgtcagtatacagagtagctgtggatccctgtgataggttatggtttgtggatactggactggtagaagttcttggaaatgtcagtcgtgtgcggcctaccactttagtaatgatggatttgactactgataaaatcattcgatcattccaaatccctgcagaccaaatccggccaaccagtggtttagctagtgtaaccgtagatgtaactaaagacacctgtgatgatgcctatgcgtatatacctgatttaggaggatatggacttatagtgtacagcctaagggaagacagatcctggagaataactcacaactatttctacttggaacctctagctggagagttcgatatctccggtcaccgttttcaatggaacgacggtatattcagtgttgaactaactaatgttaaaccagatggaaatagagacttgtatttccattccatggcaggaacgcatatgtatcgagtgcctacgaacattgtaaagaaccacactttagctacgaggtcataccatggagaggatttccagaatcttggagatcgtggagccggatctcagacctcttccgcagacatccacaaacccagcgg

898 bp

F1: acgctggggctgggggtagta

R1: ccgctgggtttgtggatgtctg

Frame+3

Amino acid

AGAGGSSSPIIYEDPNLPNSGWNPSEGIDYNHVTNIPMGANVWRNKLFITVPRRRLGVPSTLNYVPLDSPRRHNVPLIPYPDWDTNIYPDPKNSGDNFVSVYRVAVDPCDRLWFVDTGLVEVLGNVSRVRPTTLVMMDLTTDKIIRSFQIPADQIRPTSGLASVTVDVTKDTCDDAYAYIPDLGGYGLIVYSLREDRSWRITHNYFYLEPLAGEFDISGHRFQWNDGIFSVELTNVKPDGNRDLYFHSMAGTHMYRVPTNIVKNHTLATRSYHGEDFQNLGDRGAGSQTSSADIHKPSG

**>laccase2**

tgtcaatgcgtgctagctgacggtgtcgagaggggtcttctaacagccaatcgcatgttacctggtcccagtattcaagtatgtgaaggtgataaggtagtaattgacgttgaaaatcacatggaaggcatggaagttactcttcactggcacggtattttccaaaaagggtctcagtattatgacggtgtgccatttgttacgcagtgccccattcaacaaggaaatacatttagatatcagtggttagctggcaatgctggtacacacttctggcacgcccatacaggattacagaagatggatggtctatacggcagcattgttatccgtcaacctccttctcacgatcccaatagccatctgtacgactatgatctcaccactcatgtagttctcataagtgactggatgcacgaagcagccgcggaaagattcccaggaagactggcggtaaatactggacaagatcctgaaagctgcttgattaacggaaaaggacaattcagagaccccaacactggctttatgaccaatacacctttggaagttttcaccatcactcctggcaaaaggtacaggttcagaatgatcaactcatttgcttcagtttgtcccgctcaattgacaatccagggtcatgacttaacgctcatcgctaccgacggtgaaccagtgcaaccagtcaaagttaacactgtcatctcattctcaggtgaacgatacgatttcgtcataaatgccgaccaagcgcccggagcctattggatccaattaagaggtttgggagagtgtggtatcagacgagttcagcaacttgctatcctgagatacgctagaggaccataccagccgtcatcgtcacctccaacctacgactttggtataagacaaggagtggtacttaatcctttggatgcaagatgtaacgaagtaagagcagacgccatttgtgtcagtcaacttaaaaatgcaaggaagattgacgaaggactcttgcaagagaggccagacgttaaaattttcttaccattcagattcttcgtctacagtcctcaagatttgtatgctcctaatacctaccagagaca

1091bp

F3: tgtcaatgcgtgctagctga

R4: tgtctctggtaggtattaggagca

Frame+1

Amino acid

CQCVLADGVERGLLTANRMLPGPSIQVCEGDKVVIDVENHMEGMEVTLHWHGIFQKGSQYYDGVPFVTQCPIQQGNTFRYQWLAGNAGTHFWHAHTGLQKMDGLYGSIVIRQPPSHDPNSHLYDYDLTTHVVLISDWMHEAAAERFPGRLAVNTGQDPESCLINGKGQFRDPNTGFMTNTPLEVFTITPGKRYRFRMINSFASVCPAQLTIQGHDLTLIATDGEPVQPVKVNTVISFSGERYDFVINADQAPGAYWIQLRGLGECGIRRVQQLAILRYARGPYQPSSSPPTYDFGIRQGVVLNPLDARCNEVRADAICVSQLKNARKIDEGLLQERPDVKIFLPFRFFVYSPQDLYAPNTYQR

**>ebony**

tggcagtttagaagattcccttatagcccttccgagaaagtatgtgttttcaaaacagctctaacattcgttgacagcatagcggaaatttggggaccactattgaatggaagggctattctcgtgattccaaagtcggttactaaagatccagaaacatttgtttctgttttggaaaactataagattgagcggttaattttggtaccctcacttttaagaaaccttcttatgtttttacaaatgagaaaagaagatttgttactcagaaatttaaagacatgggtctgttcgggaggaacccttgtagtttctttggtcgaagagtttttcaagtactttcctccaaacgagtaccagctttgcaacttttacggcagcaccgaaataatgggcgacgtcacttatcacagtatcacaaattctgaacaactgaagcacataaacaaagtgccaataggtgcacccctggataatacgatcctttaccttctggacaaagatttccggccggtaaaagcgggtgatatcggtgaactgttcgtttccggtttgaatctggccagtggttacgtcagcggaagagatccggaaaaattcctcgacaatccattagcaatcgatcccacctacgggaaactgtaccgtacgggggacttcgcccgtctagagaagggcatacttttctacgaaggccgcaccgattctcaggtgaaaattagaggacacagagtagatacagctgaggtggagaaagcagtcaattctttggaagcagttgaaaaaggtgtggtgctatgttacaaaccaggagaaatgaaccaagctcttcttgctttcatcacaactgagagtccgataagtgaacaccaaatcgagctcatgcttaaagaaaaattagcgtcgtacatggttccacaagttatactggtggaaaaaattcctttattagtgaacggcaaaattgaccgacagtatcttttaaaatcgtacgaaaacaccaacaataacgaagaccttgagccagcaattgacatcagctacgaaggagtaccgatccatcaaatagaagcagcgaaagtactctttgaaacggtagcttgtgtactcaatcgagctgtcaggtctcaaatttccattaattcaaacttctttgaaattggaggaaactccctaaattctatatataccattccaaaatgagtgaacaagg

1213 bp

F2: tggcagtttagaagattccctta

R2: ccttgttcactcattttggaatgg

Frame+1

Amino acid

WQFRRFPYSPSEKVCVFKTALTFVDSIAEIWGPLLNGRAILVIPKSVTKDPETFVSVLENYKIERLILVPSLLRNLLMFLQMRKEDLLLRNLKTWVCSGGTLVVSLVEEFFKYFPPNEYQLCNFYGSTEIMGDVTYHSITNSEQLKHINKVPIGAPLDNTILYLLDKDFRPVKAGDIGELFVSGLNLASGYVSGRDPEKFLDNPLAIDPTYGKLYRTGDFARLEKGILFYEGRTDSQVKIRGHRVDTAEVEKAVNSLEAVEKGVVLCYKPGEMNQALLAFITTESPISEHQIELMLKEKLASYMVPQVILVEKIPLLVNGKIDRQYLLKSYENTNNNEDLEPAIDISYEGVPIHQIEAAKVLFETVACVLNRAVRSQISINSNFFEIGGNSLNSIYTIPK*VNK

**>Ago2**

tcggcaagtacatgaagcagttaagagttgtttatgaaattccaaatttgcccagttctagaagaagttataaggttaacggtataaatgacccaccggccgtgaaaacctttaaagatgcgaataatagagaaattaccatccaacggtattttgaaacagaaaaaaggtgcaaattaagatatcctcagatgccgacactatgggtcggatcttcagctagatcggataatcctatacttgtacctattgaattgtgtacaatagaagataatcagacaattaatagaaaaatgacagaaggacagacaagaaacatgataagatatgctgcaacctctactacagtacgcaagaataagattatggaaggaataacacgggctaattttaacaaccatcccactgtacgagaattcggtttttcagtttctagcgcatttgaaaaattagatgccagaatcttgccccctcccagattaggctatgccggtaaagaagtgaatgttgataaaggaatatggagaggtgacaaattcttccaagcggtcacgattaacaaatggacaatcgtgtgcgctgataggaggccaccaagaccagatgacttacggaacttagcttcacaattacttagagaagcacgcggaagcggtatgcagattggtgaagcagaacaaccattctgtacaattggtgacagaaatatggacatcaagaaatacttcacatctgttaaaggcaaatatgatgtaatctttgtcgtcgtaccaaatagcggaccacaatatagttacgttaaaacagcagctgagattaatgtaggctgtttaacacaatgtgttaaagtaagaactgtgttaaaaatgaattctcaaacggcgttaaaccttttacttaaggttaatgccaagttaaatggtactaatcattttctatctacccgtccgcccattttgaatagaccaaccatgatcatgggcgctgacgtcactcatcccagccctgattcgcaacatataccgagtgtggcggcagtcactgcatcttatgatccaaaagcctttaagtacaatatctgctggagattgcaaccacccagacaggagat

1111 bp

F1: tcggcaagtacatgaagcag

R1: atctcctgtctgggtggttg

Frame+3

Amino acid

GKYMKQLRVVYEIPNLPSSRRSYKVNGINDPPAVKTFKDANNREITIQRYFETEKRCKLRYPQMPTLWVGSSARSDNPILVPIELCTIEDNQTINRKMTEGQTRNMIRYAATSTTVRKNKIMEGITRANFNNHPTVREFGFSVSSAFEKLDARILPPPRLGYAGKEVNVDKGIWRGDKFFQAVTINKWTIVCADRRPPRPDDLRNLASQLLREARGSGMQIGEAEQPFCTIGDRNMDIKKYFTSVKGKYDVIFVVVPNSGPQYSYVKTAAEINVGCLTQCVKVRTVLKMNSQTALNLLLKVNAKLNGTNHFLSTRPPILNRPTMIMGADVTHPSPDSQHIPSVAAVTASYDPKAFKYNICWRLQPPRQE

**>Dcr2**

ggttgcacacgtatctgctcatgatgaatatgcagctgcaaaaactaatagataggttcgataacttcatggcccaaaagaactatataatagatgatgaagttttgatattactccaggaggatgaataccaactggcagaatcggtagatgtacctaaggtacttggtgatattttcgaagcagtagctggtgctatttatttggatagtaaaaaatgtctgaaaacggtgtgggaagtgttttataagataatgtggaaagaaattagtttattttctagtaatattccgaaaaatgcgataagaaggctatttgaatggacttccgctcatccta

339 bp

F2: ggttgcacacgtatctgctc

R2: taggatgagcggaagtccat

Frame+3

Amino acid

LHTYLLMMNMQLQKLIDRFDNFMAQKNYIIDDEVLILLQEDEYQLAESVDVPKVLGDIFEAVAGAIYLDSKKCLKTVWEVFYKIMWKEISLFSSNIPKNAIRRLFEWTSAHP

**>silC**

ggattcaaagtgatgcagatgttaccgccccccttgatgattgtagcaagacagaaaaaagaaatgatttcttggcagctgcccctagaaatagaaggcacaaatggcgaaacaaatgaaaattatacatctagaactatgtgccatgatattattaaacaatatagatttgcaggaagtaaacagaaattaaaacaagagaatgtcattgtttctgttactacctcatcagtaaccaatgtaagcttcactataagggtagacaatcaaaaggattttactctggaattaaataaagaaatagagtttgacattactccaagcggaccaagatactttttctacaatttcactagcaatgatacgttgttgagcaaaggagactccaattatgaaaccgtaattctggaagtgacctcagaggatgacatttgcatgactgtcagtatacaaaacatcagttgtccagtgtttgatacaaaccaagatgttacttttagaggcttttacgaaactgttaatagaaaaggagggatgaatataccgaaatataaattccctcatggattttatgtcgtctttgtagctaagcctgatgattatgcctgtgataagggttccggaaatttagacgccgttacaaatccagatcgactgaaaagcattaagttagtaattaagccaagtattacctactctgattatgtcaaagctgtgctttttactttgggatctgtaggagcattttacataatattcggacttccgtacttcatatactcggtgaaaaaatctctgcctagggaaatggcgtatgttgatggtaacttcccaacaactcccagtgcagaaatgacctccgtacagcgcaccgtttcggccgttagtggcccttcagtggacatggccgacttcgataccttggccgaggtggacaccgatagggacctgaggttgggtaggggagagccttatttggtggatttggcgaggaagcatcccaaagagttgacgaggaaatcgtatttgtatctgtacaatgttgtcaccgttgccattttctacgctttaccagtgatacagttggtcatcacttatcaaagggttttgaatgagacgggccaacaagacttgtgttattacaatttcttatgtgcacatccattaggtgtactgggagatttcaaccacgtgttttcgaacataggatatgtcctattgggagttctattcctaataattacctacttaagagaactttcgcacaaagacgatgatttcgaccggcaatttggcattccccaacattatggattgttttacgccatgggtgtggccttgatcatggagggtgttttgagcgcaagttaccatgtctgtcccaacttcttgaacttccaatttgattcgagctttatgtacgtcatggcagtgcttgttatggtaaagttgtaccaaaatcgacatccagatataaacgccaacgcttacactacatttggagtactagcagttgctgtcgtcttagctacgattggactactcgaaggaaatgtctatttttggacaatattcgtgattatgcacatcctgatgtgcttctacttaagcgttaaggtctactacatgggatgctggagtgtgcgagatatatcgatgcagaagttccgtcaagtttggatctacgatttttggtctggaccagtaaatgtcataaaaccgtgccataaagctagattcgtgcttctcttcttgggcaacttgtgcaattggggcttggctatcttcgccatctataaacttcctaagaactttccagtcttccttttggctatatttatggccaatactctcctctatttcgtcttctatatcgtcatgaagtacattaacaaagaacatgtaagaattttaacgtggaatcttcctttttatgtcaacgttgtgtgcagtatctgccatgtggttcttcttacataaagcaatatcttggaagaaaacggcggcacaatcgagacaattcaacgtagaatgcaagctgttccatttctacgactcccatgacatttggcactttct

2079 bp

Frame+1

GFKVMQMLPPPLMIVARQKKEMISWQLPLEIEGTNGETNENYTSRTMCHDIIKQYRFAGSKQKLKQENVIVSVTTSSVTNVSFTIRVDNQKDFTLELNKEIEFDITPSGPRYFFYNFTSNDTLLSKGDSNYETVILEVTSEDDICMTVSIQNISCPVFDTNQDVTFRGFYETVNRKGGMNIPKYKFPHGFYVVFVAKPDDYACDKGSGNLDAVTNPDRLKSIKLVIKPSITYSDYVKAVLFTLGSVGAFYIIFGLPYFIYSVKKSLPREMAYVDGNFPTTPSAEMTSVQRTVSAVSGPSVDMADFDTLAEVDTDRDLRLGRGEPYLVDLARKHPKELTRKSYLYLYNVVTVAIFYALPVIQLVITYQRVLNETGQQDLCYYNFLCAHPLGVLGDFNHVFSNIGYVLLGVLFLIITYLRELSHKDDDFDRQFGIPQHYGLFYAMGVALIMEGVLSASYHVCPNFLNFQFDSSFMYVMAVLVMVKLYQNRHPDINANAYTTFGVLAVAVVLATIGLLEGNVYFWTIFVIMHILMCFYLSVKVYYMGCWSVRDISMQKFRQVWIYDFWSGPVNVIKPCHKARFVLLFLGNLCNWGLAIFAIYKLPKNFPVFLLAIFMANTLLYFVFYIVMKYINKEHVRILTWNLPFYVNVVCSICHVVLLT*SNILEENGGTIETIQRRMQAVPFLRLP*HLALS

**>silA**

gaagagtacgacatagcgttaaaaggtgctgttatgaaatttgatatcaccatgaaaaatttgcaatggattctgcttgcagtttgttttggtttaataaattgtaaatcaactaataatctgacaattgaacaacagcttttaaccaatggaagtcccatcacaaatcaacttaacgcaaacaaacaaattattttattatatcgaaactttacgtcgataaacccctacaggataatcacatcttccgagaatgccaagaaggaatttcccgttttgattgttgctgaacagagaactcaagtgacagcatggtctattccgatgcttatcgaatcgactaaaccaggagtagaatactcttataatatgtcctctaaaacattgtgtcatgattatatgaacattataattctttctccaggaaatgatagtacgccctttatatttccaacaaaatttcatagtggcattgtcaacggcgagcacaactgatgtaaacgtaactgtggaattgaaagaagagccggatttttacctaatacaagataaaccgtataacatgacggtgggacccagtgagccaagatatgcttatttcagattcgaaaaggacactgctgacacggtcgtcatcgaagtagattcagaagacgaactctgtcttacaatttctgtccaggacagcaagtgcccggtttttgacaataacaaagatgtaaaatatgagggcatccatcaaacaatcaatacaaaaggtgctattactttattgaaacgcagttacaaagatggattcttccttgttttcgtcgcgaaaccagatacctatgactgcagtcaggaaagctcaaatctaccgagactgacaagaaccgtttccatagagcagatggttcgcacacatgttaactttattatcagaaatagcattacagcaaatgattacactatcgctgtaggtgctacatttttaatattaatcggttccggtatcctggtgactgtagctgcgcttatcttccataggtatgggaccattgctagaaataagtacgaagatgtcatagtatcggactattttgaagctttgtctgaagaacagatcagctcattgctgaggaaacttgatctacatgtgtccgaattatcaagacatcctaatagaatcaaaaaaaggtcttacaactatttgtatcatacgttgagtatcgccatattttacagcatacctgtagttcagctagttttcacctaccagaggatcgttaatcgcactggagatgaagacatgtgttactataacttcctatgcgcacatcctgcctttcgtttcagcgacttcaatcatattttctccaatatcggctacgttattttcggcatcattttcatttgtgtagttgctgacaggcatagaataatcaagttgagaaaggacaaaggaataccagtgcattatggaatatttcatgctatgggcgtggctttaattattgaagggttgttgtcagcatgttaccatatttgcccgagtcagtccaattatcaattcgatacaagttttatgtacattatggcagttttatgcatggtaaagttataccagaaccgtcatccagatattaatgccactgcgtacacaacgtttactgttttgggatgtgccatatttatggctatgattggaattttgaacggaaacttagccatctggatcatcttcgtagtatgctacacgttgctctgtttatttttatcattaaaaatttatttccttaactacgtcgtcgatggattaaatcagtttcgaagttccgtcaccaagaaaggttttactcaacacgcacttaaacctatacggaaagctagatttataatactgctgatagcgaatatagcgaattattcaatgttgatttgtgggctcttgttgtatagcgacaacgtcactgactttggaactttcttgttagcgttattgatgggtaacagcgtcattcattgtgtgttttatacgtgtatgaagctgataagcaaagaaaaaatttgttatgaagcaattctgtatggggttctcgcaatagtgtgttggggttcatcaagtgtatttttcttagatgcagcaactttatggacggtcacacctgcagaatctcgtcaatggaaccaagaatgtatagtcttcaagttcttcgataaacacgatatttggcacctactgagtgccccagctctctattttacttttatgtatttgatgtctttggacgatgacatacttgatgtagaacaaagagatctacaagtattctagattatgggtttaaatttgcagaagaatactgttctaaaccaatattaatttgatatattggtatttgtaggatttaataaactaaaacaggattgacttttgacctattgtggatgttaagacagtgtacctttatcgctgtaatttgaccgattttgatctataccgatttattggtgaggtctatttgtaccgatgtaataacttaagactatcaatcagtcagcaacgaaactcctcttgtgccttctacacttatctgttcgttgctagttgttgtcaatatctcacattctgactttgatgttatttagctacatttctccaataaacaatatttcttgtccaactttcattcttgttatgccatgtatcatac

2696 bp

Frame+2

Amino acid

KSTT*R*KVLL*NLISP*KICNGFCLQFVLV**IVNQLII*QLNNSF*PMEVPSQINLTQTNKLFYYIETLRR*TPTG*SHLPRMPRRNFPF*LLLNRELK*QHGLFRCLSNRLNQE*NTLIICPLKHCVMII*TL*FFLQEMIVRPLYFQQNFIVALSTASTTDVNVTVELKEEPDFYLIQDKPYNMTVGPSEPRYAYFRFEKDTADTVVIEVDSEDELCLTISVQDSKCPVFDNNKDVKYEGIHQTINTKGAITLLKRSYKDGFFLVFVAKPDTYDCSQESSNLPRLTRTVSIEQMVRTHVNFIIRNSITANDYTIAVGATFLILIGSGILVTVAALIFHRYGTIARNKYEDVIVSDYFEALSEEQISSLLRKLDLHVSELSRHPNRIKKRSYNYLYHTLSIAIFYSIPVVQLVFTYQRIVNRTGDEDMCYYNFLCAHPAFRFSDFNHIFSNIGYVIFGIIFICVVADRHRIIKLRKDKGIPVHYGIFHAMGVALIIEGLLSACYHICPSQSNYQFDTSFMYIMAVLCMVKLYQNRHPDINATAYTTFTVLGCAIFMAMIGILNGNLAIWIIFVVCYTLLCLFLSLKIYFLNYVVDGLNQFRSSVTKKGFTQHALKPIRKARFIILLIANIANYSMLICGLLLYSDNVTDFGTFLLALLMGNSVIHCVFYTCMKLISKEKICYEAILYGVLAIVCWGSSSVFFLDAATLWTVTPAESRQWNQECIVFKFFDKHDIWHLLSAPALYFTFMYLMSLDDDILDVEQRDLQVF*IMGLNLQKNTVLNQY*FDILVFVGFNKLKQD*LLTYCGC*DSVPLSL*FDRF*SIPIYW*GLFVPM**LKTINQSATKLLLCLLHLSVRC*LLSISHILTLMLFSYISPINNISCPTFILVMPCIIX
